# Severe maternal morbidity: A population-based study of an expanded measure and associated factors

**DOI:** 10.1371/journal.pone.0182343

**Published:** 2017-08-07

**Authors:** Victoria Lazariu, Trang Nguyen, Louise-Anne McNutt, Jillian Jeffrey, Marilyn Kacica

**Affiliations:** 1 Division of Family Health, Center for Community Health, New York State Department of Health, Albany, New York, United States of America; 2 Department of Epidemiology and Biostatistics, University at Albany, State University of New York, Albany, New York, United States of America; 3 Office of Public Health, New York State Department of Health, Albany, New York, United States of America; 4 Institute for Health and the Environment, University at Albany, State University of New York, Albany, New York, United States of America; University of Washington, UNITED STATES

## Abstract

Severe maternal morbidity conditions such as sepsis, embolism and cardiac arrest during the delivery hospitalization period can lead to extended length of hospital stays, life-long maternal health problems, and high medical costs. Most importantly, these conditions also contribute to the risk of maternal death. This population-based observational study proposed and evaluated the impact of expanding the Centers for Disease Control and Prevention (CDC) measure of severe maternal morbidity by including additional comorbidities and intensive care admissions during delivery hospitalizations and examined associated factors. A New York State linked hospitalization and birth record database was used. Study participants included all New York State female residents, ages 10 to 55 years, who delivered a live infant in a New York acute care hospital between 2008 and 2013, inclusive. Incidence trends for both severe maternal morbidity measures were evaluated longitudinally. Associations between covariates and the two severe maternal morbidity measures were examined with logistic regression models, solved using generalized estimating equations and stratified by method of delivery. The New York expanded severe maternal morbidity measure identified 34,478 cases among 1,352,600 hospital deliveries (estimated incidence 2.55%) representing a 3% increase in the number of cases compared to the CDC measure. Both estimates increased over the study period (p<0.001). Covariates with an odds ratio > 1.5 included most measured comorbidities (e.g., pregnancy-induced hypertension, placentation disorder), multiple births, preterm birth, no prenatal care, hospitalization prior to delivery, higher levels of perinatal care birthing facilities and race/ethnicity. Expanding the measure for severe maternal morbidity during delivery to capture intensive care admissions provides a more sensitive estimate of disease burden. Perinatal regionalization in New York appears effective in routing high risk pregnancies to higher levels of perinatal care birthing facilities.

## Introduction

Severe maternal morbidity during the delivery hospitalization is increasing in incidence[1] and is a substantial risk factor for maternal death.[1, 2] The serious medical complications which define severe maternal morbidity, such as sepsis, embolism, and cardiac arrest[1, 3], accrue substantial medical costs due to prolonged hospital stays, chronic health problems, physical and psychological stress disorders, and increased risk of maternal death.[4] The New York maternal mortality rate per 100,000 live births (17.9) is slightly below the national average (18.5)[5] but far exceeds the Healthy People 2020 goal of 11.4. In addition, the maternal mortality rate among Black non-Hispanic women is three times higher than White non-Hispanic women in New York.[5] Efforts to address disparities in maternal mortality must include a more comprehensive understanding of its sentinel indicator, severe maternal morbidity.

The few population-based studies of severe maternal morbidity during delivery conducted in the United States have identified the following risk factors[3, 6–9]: extremes of maternal age and weight, non-White race, preexisting conditions, multiple gestations, cesarean delivery, and geographic region. The literature suggests a more robust measure would include additional preexisting conditions and admission to an intensive care unit.[10–14]

Standardized surveillance criteria for monitoring severe maternal morbidity should be deployed at the population level to inform prenatal interventions.[3, 7] This study had two purposes: (1) evaluate the impact of expanding the existing Centers for Disease Control and Prevention (CDC) measure[1] to include preexisting conditions and intensive care unit admission, and (2) identify factors associated with severe maternal morbidity. The incidence of severe maternal morbidity during delivery for both the CDC and New York measures is presented, as are associated risk factors.

## Methods

### Population

The study population included New York State female residents, ages 10 to 55 years, delivering live infants in a New York acute care, state-regulated hospital between 2008 and 2013 inclusive. Deliveries occurring outside of hospitals, in hospitals under federal jurisdiction (e.g. Indian Health services), or resulting in stillborn infants, abortions, ectopic and molar pregnancies were not included.

The study was approved and continuously monitored by the New York State Department of Health Institutional Review Board. Informed consent is not required for health services research of administrative health records. No contact occurred with women included in these analyses.

### Data sources

At the population level, identification of severe maternal morbidity relied solely on the information available in administrative database records, such as hospital discharge (S1 Document) and vital event records (birth certificates, S2 and S3 Documents). Delivery hospitalizations were identified from hospital records using an enhanced method described previously[15] and then linked to birth certificate records. The database was restricted to New York residents.

### Measurement of severe maternal morbidity during delivery

**Fig 1:**
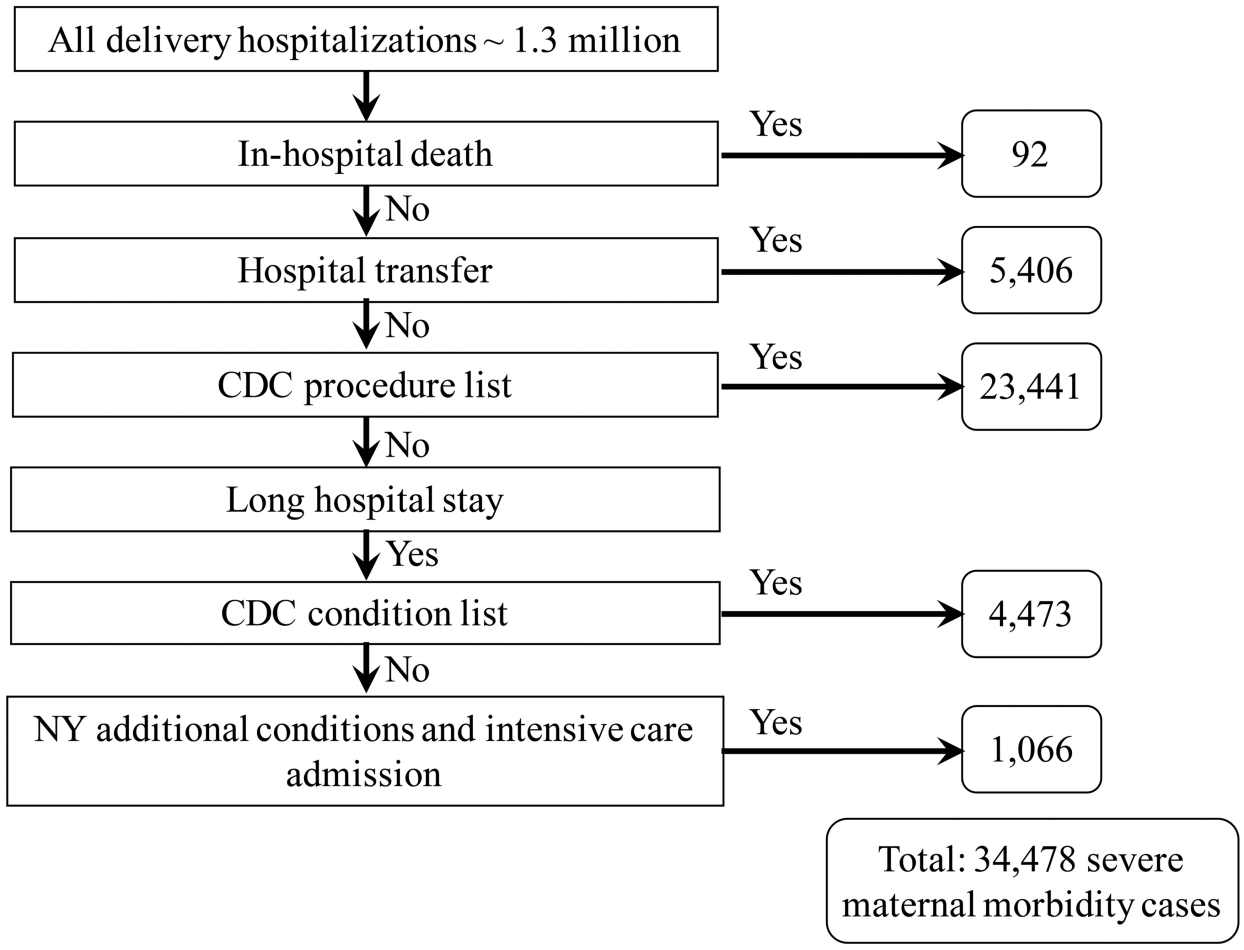
New York severe maternal morbidity measure, New York hospital deliveries 2008–2013.

The CDC measure is defined using an enhanced hierarchical algorithm with four steps (Fig 1): (1) maternal death, (2) maternal transfer from another care facility, (3) a specified list of procedures, and (4) a specified list of conditions combined with a long hospital stay.[1] A long length of stay was defined as the length of a hospital stay at or above the 90^th^ percentile for all deliveries stratified by delivery method (i.e., vaginal: three days or more, primary cesarean: five days or more, and repeat cesarean: four days or more).

The New York measure of severe maternal morbidity during delivery expands the CDC measure based on a review of the literature to include additional conditions if (1) the mother had a long length of stay in the hospital (as defined above), and (2) had an accommodation code for admission to an intensive care unit recorded on the hospital record.[10–14, 16] The additional conditions included groups of diagnostic codes: severe hypertension[10, 16], early pregnancy and postpartum hemorrhage[16], anemia[16], acute liver disease[11], complications indicative of acute heart[10, 16] or respiratory failure[10, 11], puerperal cerebrovascular disorders[11], thrombocytopenia[10, 12], sepsis[11], shock[11], pulmonary edema[10, 11, 16], venous thromboembolism[16], disseminated intravascular coagulation[10, 11], coma[11, 13, 16], delirium[11, 16], status asthmaticus[11] and status epilepticus.[11] (Table 1)

**Table 1:** New York severe maternal morbidity measurement: Indicators of severe maternal morbidity.

Severe Maternal Morbidity	International Classification of Diseases, 9^th^ revisions, Clinical Modification codes	
	Developed by the Centers for Disease Control and Prevention	New York Supplemental codes*
Acute myocardial infarction	410	
Acute liver disease		570, 646.7
Acute renal failure	584, 669.3	
Adult respiratory distress syndrome	518.5, 528.81, 518.82, 518.84, 799.1	518.7
Embolism:		
Amniotic fluid embolism	673.1	
Thrombotic embolism	415.1, 673.0, 673.2, 673.3, 673.8	
Venous thromboembolism		453
Anemia (including sickle cell anemia with crisis)	282.62, 282.64, 282.69	280, 281, 282, 283, 284.0, 284.1, 284.8, 284.9, 285
Aneurysm	441	
Cardiac arrest/ventricular fibrillation	427.41, 427.42, 427.5	
Coma		250.2, 250.3, 251.0, 572.2, 780.01, 780.03
Diabetic ketoacidosis		250.1
Delirium		293
Disseminated intravascular coagulation	286.6, 286.9, 666.3	286.7
Eclampsia	642.6	
Hemorrhage		640.0, 640.8, 640.9, 641.1, 641.2, 641.3, 641.8, 641.9, 666^
Heart complications		415.0, 428.0, 428.21, 428.23, 428.31, 428.33, 428.41, 428.43, 428.9
Heart failure during procedure or surgery	669.4, 997.1	
Internal injuries of thorax, abdomen, and pelvis	860–869	
Intracranial injuries	800, 801, 803, 804, 851–854	
Liver and biliary tract disorders in pregnancy		646.7
Oliguria		788.5, 646.2, 997.5
Puerperal cerebrovascular disorders	430, 431, 432, 433, 434, 436, 437, 671.5, 674.0, 997.2, 999.2	325, 346.6, 348.1, 348.3, 348.5, 997.01, 997.02
Pulmonary edema	428.1, 518.4	514
Sepsis	038, 995.91, 995.92, 670.2	112.5, 659.3
Severe anesthesia complications	668.0, 668.1, 668.2	
Shock	669.1, 785.5, 995.0, 995.4, 998.0	995.94, 999.4
Status asthmaticus		493.01, 493.11, 493.21, 493.91
Status epilepticus		345.3
Thrombocytopenia		287.3–5, 287.8–9, 446.6, 289.84
Thyrotoxic crisis		242
Uterine rupture		665.0, 665.1, 665.5, 665.7–9
Urea and creatine		270.6, 790.5
Procedure codes		
Blood transfusion	99.0	
Cardio monitoring	89.6	
Conversion of cardiac rhythm	99.6	
Hysterectomy	68.3–68.9	
Operations on heart and pericardium	35, 36, 37, 39.	
Temporary tracheostomy	31.1	
Ventilation	93.9, 96.01–96.05, 96.7	

*Records with these codes were classified as severe maternal morbidity conditional on longer hospital stay and admission to intensive care.

^ One code in this subgroup (666.3) is included in the CDC list of indicators of severe maternal morbidity.

### Covariates

An extensive list of covariates including maternal characteristics, medical history and hospital-related factors were extracted from the linked database. The maternal characteristics included age, race, ethnicity, education, place of birth (United States or outside United States) and residence location (New York City vs rest of state), primary payer for delivery, employment during pregnancy, inferred marital status (based on presence of completed paternity affidavit), smoking, drinking, and illegal drug use. Medical history included pre-pregnancy weight status and weight gain during pregnancy, prenatal care received and type of provider, self-reported depression during pregnancy, type of delivery (vaginal delivery vs primary or repeat cesarean section), hospitalizations during pregnancy, and number of previous births (parity). Pre-pregnancy body mass index was derived from the pre-pregnancy weight and height recorded in the birth record. The body mass index was defined as maternal weight divided by height squared; body mass index was used to derive the weight status (underweight, normal, overweight and obese) and to assess the amount of weight gained during pregnancy in relation to recommendations.[17] The adequacy of prenatal care[18] was evaluated based on number of prenatal visits, the trimester of pregnancy for the first visit, and clinician’s estimate of gestational age. Preterm labor, fetal presentation, and plurality were included as clinical factors relevant to current pregnancy. An indicator of pregnancy hospitalizations was derived when there was at least one inpatient hospital admission within 30 days before delivery admission date. The preexisting comorbidities derived from hospital discharge records included a comprehensive list of groups of diagnoses previously used in other severe maternal morbidity studies.[6, 7]

Hospital-related factors consisted of day of admission for delivery (week day vs weekend), and hospital-designated level of perinatal care. In New York, the hospital level of perinatal care is designated according to availability of sub-specialty consultation, qualifications of staff, types of equipment available and volume of high-risk perinatal patients.[19]

### Analysis

Two analyses were conducted, the first to describe longitudinal trends in severe maternal morbidity and the second to identify associations between potential risk factors and severe maternal morbidity. The trends in annual incidence of severe maternal morbidity were described with logistic regression models, separately for the NY and CDC measures, for all deliveries, and stratified by delivery method. In the second analysis, associations between covariates and each of the severe maternal morbidity measures were assessed using logistic regression modeling. All logistic regression models were solved using generalized estimating equations (GEE)[20] to account for correlations of care for pregnant women within the same delivery hospital and for women with separate distinct pregnancies during the study period. We used the quasi-likelihood information criterion[20] with a parameter penalty to compare models. Effect modification was addressed by conducting separate regression analysis by delivery mode (i.e., vaginal delivery, cesarean section). With the exception of the comorbidities, the other covariates were selected into the regression model based on p-values. Additionally, confounding was assessed; if a factor had a p-value>0.05 but was a confounder for any other factor in the model, it was retained.

Missing values for all covariates were imputed through a sequential regression for multiple imputation.[21] This Bayesian approach, which uses all the variables in the analyses and assumes data is missing at random, preserves the existing correlations among covariates and the interdependencies in the data.[22] A total of five completed datasets were generated and the results from the regression analyses were pooled across the imputed datasets. Missing data imputations were performed with IVEware software, [21] and all other analyses were performed using SAS^®^ version 9.4 (SAS Institute, Cary, NC).

## Results

A total of 1,352,600 hospital delivery records were linked to live births among New York residents between 2008 and 2013 (93% of live birth records) and were included in the study. The maternal demographic characteristics (such as age, race, and ethnicity) of the unlinked and linked birth records were similar.

Overall, 87% of records had complete data. The most common factors associated with missing data were self-reported depression during pregnancy (9.48%), pre-pregnancy body mass index (2.56%), and weight gain during pregnancy (2.09%). The remaining factors associated with missing data each affected less than 0.7% of the records.

### Incidence of severe maternal morbidity: CDC and New York measures

The CDC measure identified 33,412 cases of severe maternal morbidity with an estimated incidence of 2.47% (95% confidence limits (2.44, 2.49)) and the New York measure identified 34,478 cases with a slightly higher estimated incidence of 2.55% (95% confidence limits (2.52, 2.57)). The majority of severe maternal morbidity cases were captured through the list of life-saving procedures (Fig 1). The expanded definition for the New York measure identified 3% more severe maternal morbidity cases compared to the CDC measure, most of these additional deliveries were cesarean deliveries (primary 58% and repeat 19%).

Among the additional 1,066 cases captured only by the New York measure, the average length of stay was seven days for vaginal deliveries and ten days for cesarean deliveries. The most common diagnoses affecting these deliveries included current conditions complicating pregnancy (68%, n = 730), severe hypertension including severe pre-eclampsia (70%, n = 750), indications for care or intervention related to labor and delivery (48%, 512), early or threatened labor (51%, n = 545), abnormality of organs and soft tissues of pelvis (29%, n = 311), conditions or status of mother complicating pregnancy (19%, n = 207), and malposition or malpresentation of the fetus (19%, n = 201).

**Fig 2:**
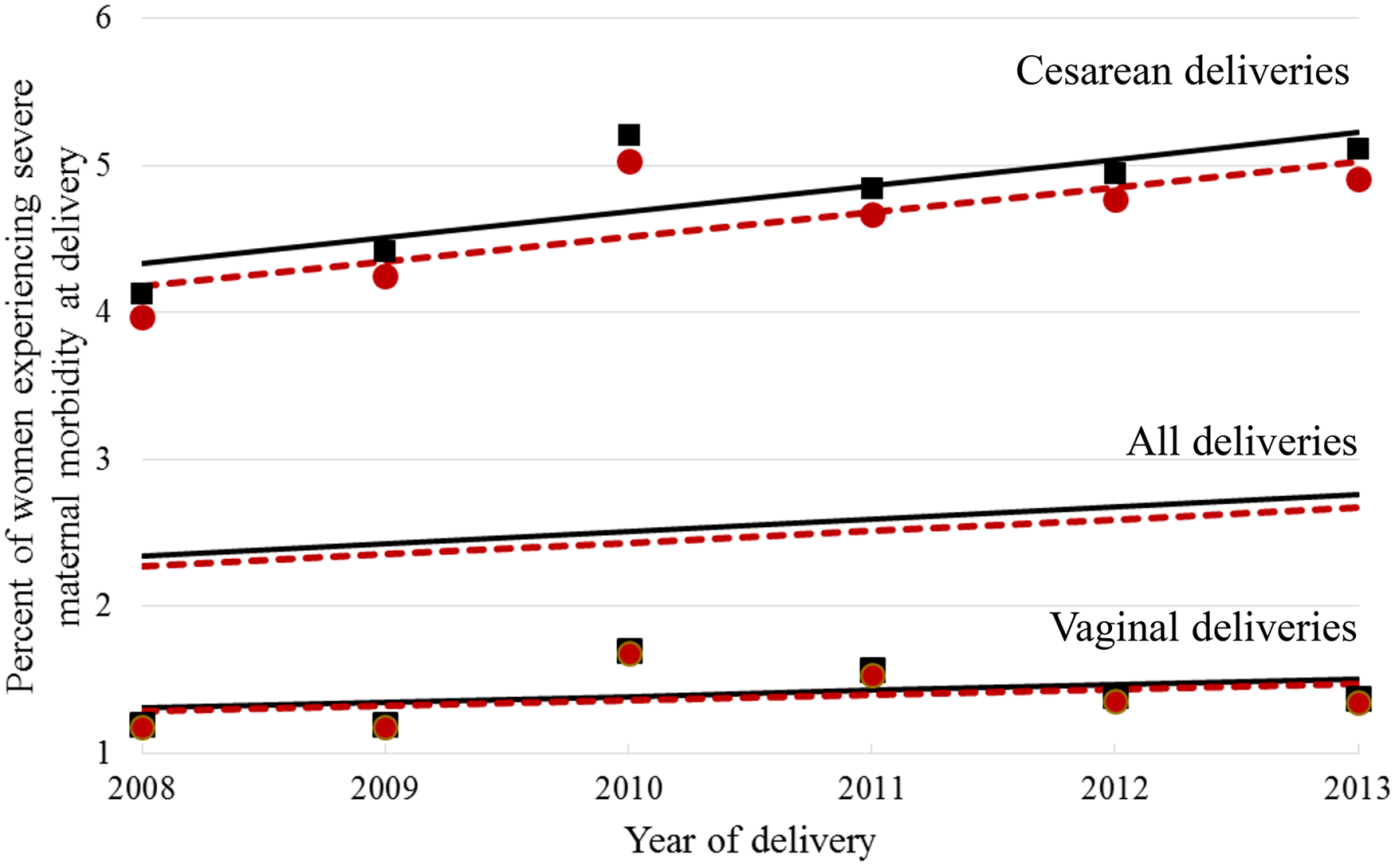
Percent of deliveries with severe maternal morbidity, New York hospital deliveries 2008–2013*. *Restricted to New York residents. CDC severe maternal morbidity definition: observed designated with a red circle, **estimated** trend designated with a red line; New York severe maternal morbidity definition: observed designated with a black square, estimated trend designated with a black line.

Using the New York measure, the percent of severe maternal morbidity deliveries increased from 2.19% in 2008 to 2.64% in 2013 (difference = 0.45%, 95% confidence limits (0.36, 0.54), Fig 2). Similar trends were observed using the CDC measure. Severe maternal morbidity was, on average, about three times greater among cesarean deliveries (4.77%) compared to vaginal deliveries (1.41%) (Fig 2). When stratified by method of delivery, the number of cases of severe maternal morbidity identified by the New York measure, compared to the CDC measure, increased slightly (by 0.18%, 95% confidence limits (0.10%, 0.26%)) among vaginal deliveries and by almost a full percent (0.99%, 95% confidence limits (0.78, 1.20)) among cesarean deliveries over the study period (Fig 2). Estimated slopes of regression lines modeling severe maternal morbidity over time were greater than zero (each with p-value < .0001).

### Association between covariates and severe maternal morbidity using all identified cases (New York measure)

The S1 and S2 Tables in the Supporting Information provides results from the bivariate and multivariate analyses, stratified by the method of delivery, for the associations between covariates and severe maternal morbidity cases identified by the New York measure. Demographic predictors of severe maternal morbidity were age younger than 20 years or older than 35 years, non-White, less educated, single and without private insurance. Clinical factors associated with greater odds of severe maternal morbidity included inadequate prenatal care, and being underweight. Difficulties with the pregnancies, identified by hospitalizations during pregnancy, preterm labor, and cesarean section were also associated with severe maternal morbidity. Finally, women delivering in regional perinatal centers and level 3 hospitals had a higher likelihood of having severe maternal morbidity.

Common delivery complications figured prominently among the severe maternal morbidity cases, including hemorrhage and anemia, as well as rare events, including heart failure, disseminated intravascular coagulation and thrombocytopenia (S3 Table). Hysterectomy, a rare procedure among deliveries, was present in over 4% of the severe maternal morbidity cases.

During the study period, rates among all deliveries for a number of conditions and procedures defining severe maternal morbidity increased temporally, including acute renal failure, shock, disseminated intravascular coagulation, pulmonary edema, blood transfusion, and hysterectomy. Decreasing rates were noted for uterine rupture, cardiac monitoring, eclampsia, and severe anesthesia complications (S3 Table).

An extensive list of possible risk factors was explored in the development of the logistic regression model (S1 and S2 Tables). The adjusted odds ratios (aOR) for severe maternal morbidity among covariates in the final models were generated for both the CDC and New York measures. Because the aOR did not differ substantively for any covariates (S1 Fig and S4 Table), we focus on the results using the New York severe maternal morbidity measure.

**Fig 3:**
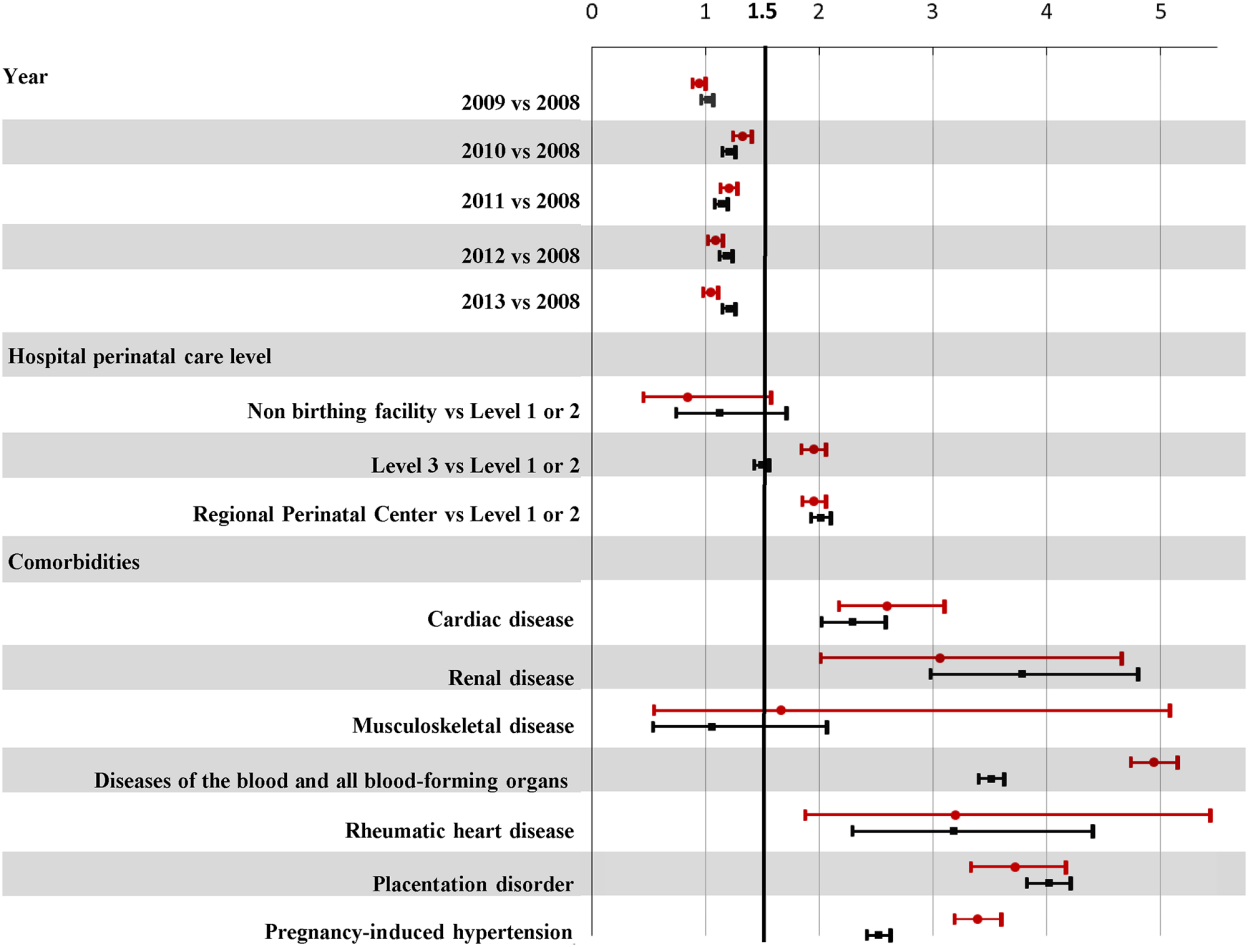
Adjusted odds ratios for severe maternal morbidity by year of delivery, hospital and comorbid conditions, stratified by method of delivery: NYS 2008–2013 delivery hospitalizations. aOR for severe maternal morbidity and 95% confidence limits: Vaginal delivery designated with a red circle; Cesarean delivery designated with a black square. aOR derived from logistic regression model adjusting for year of delivery, maternal and hospital characteristics, medical history and clinical factors, and comorbid conditions. When comorbidities were removed from the model, the findings for remaining factors did not differ substantively.

The estimated odds for severe maternal morbidity decreased since 2010 for vaginal deliveries but remained fairly constant for cesarean deliveries (Fig 3). Regionalization policies in effect in NYS were reflected in the higher likelihood (or odds) for severe maternal morbidity for both delivery methods in regional perinatal centers (level 4 hospitals), and level 3 hospitals.

**Fig 4:**
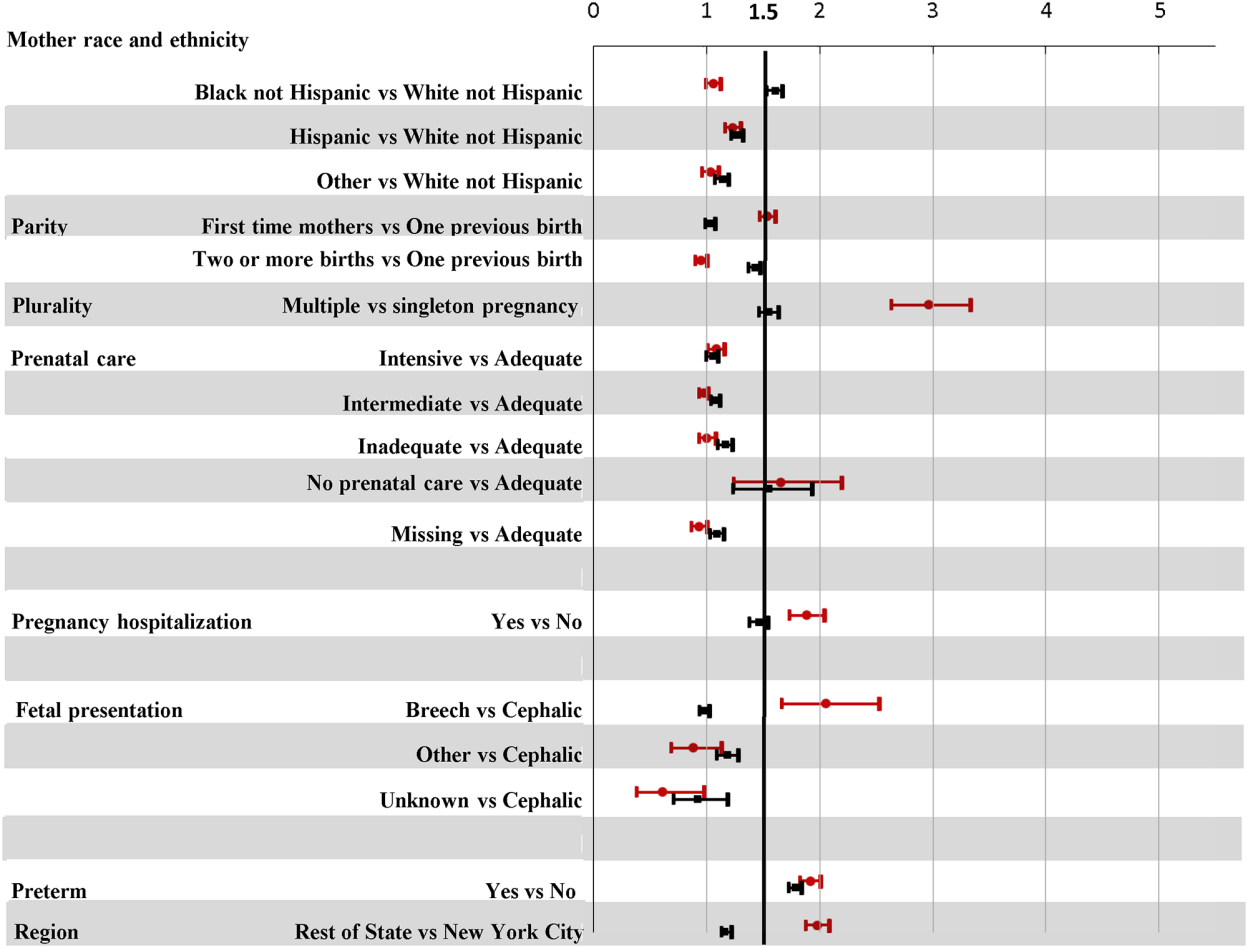
Adjusted odds ratios for severe maternal morbidity by maternal characteristics and clinical factors, stratified by method of delivery: NYS 2008–2013 delivery hospitalizations. aOR for severe maternal morbidity and 95% confidence limits: Vaginal delivery designated with a red circle; Cesarean delivery designated with a black square. aOR derived from logistic regression model adjusting for year of delivery, maternal and hospital characteristics, medical history and clinical factors, and comorbid conditions. When comorbidities were dropped from the model, the findings for remaining factors did not differ substantively.

Regardless of method of delivery, increased odds (aOR>1.5) for severe maternal morbidity were observed for women without prenatal care, with hospital admissions in the month prior to delivery, or with preterm labor (Figs 3 and 4).

As noted in other studies[23, 24], method of delivery appears to modify the associations with severe maternal morbidity. For vaginal deliveries, higher odds of severe maternal morbidity were linked to first time mothers or those with breech position. Black and White women with vaginal deliveries had similar estimated odds ratios for severe maternal morbidity; however, an elevated odds was noted for Hispanic and women of Other races when compared to Whites.

In contrast, for cesarean deliveries, a higher odds of severe maternal morbidity was linked to mothers with two or more previous deliveries and Black women. White women had the lowest odds for severe maternal morbidity (Figs 3 and 4).

The magnitudes of the aORs were similar regardless of method of delivery for all but two groups of comorbidities. Women with diseases of the blood and blood-forming organs or pregnancy-induced hypertension were less likely to experience severe maternal morbidity if they had a cesarean compared to vaginal delivery (Fig 3).

## Discussion

### Main findings

The identification and monitoring of severe maternal morbidity are important for obstetric care improvement and resource allocation. The New York measure expanded the CDC population-based measure of severe maternal morbidity to include women admitted to intensive care, an indicator of disease severity and medical care associated with substantial hospital costs.[25] Also, the list of qualifying medical conditions was expanded based on evidence suggesting they constitute severe maternal morbidity.[10–14, 16] During the study period, New York’s definition resulted in a 3% expansion in the number of cases identified compared to using the CDC definition.

The results also indicate how well a triage system works to direct high risk pregnancies to hospitals with the highest level of care.[19] Severe maternal morbidity cases were more likely to be found in level 3 and 4 hospitals and transfers to a higher level of care occurred in 15.70% of these cases (Fig 1), thus suggesting that high risk deliveries are appropriately routed to the hospitals best equipped to provide the care needed.[19]

The regional variation in levels of perinatal facilities and in the race/ethnicity of mothers explain the lower estimated odds associated with deliveries in New York City. Almost all New York City deliveries (86%) were in level 3 and 4 hospitals (compared to 44% in the rest of the state). Women were more evenly distributed across race/ethnic groups in New York City while in the rest of the state white, non-Hispanic women represented the majority of the deliveries (66%).

After adjusting for all covariates, the race/ethnic disparities in severe maternal morbidity mirrored known disparities in maternal mortality[5, 26] and morbidity[8, 27] with White non-Hispanic women experiencing the lowest odds for severe maternal morbidity at delivery. The one exception was that Black and White non-Hispanic mothers with vaginal deliveries had similar experiences.

In general, women who deliver vaginally were less likely to experience severe maternal morbidity than women with cesarean deliveries. In addition, the odds of severe maternal morbidity among women who delivered vaginally decreased since 2010, yet the rate remained stable for cesarean deliveries. This finding suggests that obstetricians have been successful in targeting low risk women for vaginal delivery. The risk factors for severe maternal morbidity depend on delivery mode (Figs 3 and 4): lower odds are associated with cesarean deliveries for deliveries of multiple infants or for pregnancies with hospital admissions before delivery.

### Strengths and limitations

Methodological caveats unique to this study may impact interpretations. The identification of severe maternal morbidity was based on hospital discharge records. Coding practices vary by hospital, thus the accuracy and completeness of the hospital discharge data will also vary. The published literature suggests that intensive care is more accurately recorded[28, 29] in hospital discharge data than pre-existing maternal medical conditions and complications of pregnancy.[30] Missing data was assumed to be random. Measurement of severe maternal morbidity and the definition of most comorbid conditions were based on diagnosis codes present in the hospital discharge records. A total of 625 records were classified as severe maternal morbidity based solely on diagnosis codes used also in the definition of comorbid conditions (including a longer hospital stay and an admission to intensive care). The exclusion of these records had no substantive impact on the results of the analyses presented here.

Similar to the CDC measure, this expanded measure of severe maternal morbidity can be generalized beyond New York hospital discharge data. All the elements needed in the hierarchical algorithm, including admission to intensive care, are available in other databases used for maternal health studies and surveillance such as the inpatient discharge national samples available from the Healthcare Cost and Utilization Project.[25, 31]

The role of intensive care in identification of severe maternal morbidity needs further exploration since almost a third of deliveries with intensive care were not classified as severe maternal morbidity using either measure because they did not meet the hospital length of stay or diagnoses and procedure criteria. Other studies used transfer to intensive care as an independent criterion for severe maternal morbidity with similar findings regarding common risk factors.[9]

## Conclusions

This population-based study of severe maternal morbidity at the time of delivery suggests that expanding the definition of severe maternal morbidity may identify additional appropriate cases. At the population level, improved measurement of severe maternal morbidity is instrumental for better monitoring and resource planning. For instance, the clustering of cases in level 3 and 4 hospitals suggests that regionalization successfully triages maternal care.

## Supporting information

S1 Document Sparcs_dgc_manual. (PDF)

S2 Document APPM 500.0. (PDF)

S3 Document DOH-4269 VR data application (Word form). (DOC)

S1 Table Demographic, clinical and hospital-related characteristics: New York hospital vaginal deliveries and New York severe maternal morbidity algorithm, 2008–2013 (n = 893,497). (DOCX)

S2 Table Demographic, clinical and hospital-related characteristics: New York cesarean deliveries and New York severe maternal morbidity algorithm, 2008–2013 (n = 459,103). (DOCX)

S3 Table Percent of selected complications and procedures during delivery hospitalizations: New York 2008–2013 (n = 1,352,600). (DOCX)

S4 Table Comparison of New York and CDC measures for severe maternal morbidity: Adjusted odds ratios and 95% confidence limits. (DOCX)

S1 Fig Comparison of adjusted odds ratios using New York and CDC measures for severe maternal morbidity. Blue line: CDC SMM measure; red line: NY SMM measure.
(TIF)

## References

[1] Callaghan WM, Creanga AA, Kuklina EV. Severe maternal morbidity among delivery and postpartum hospitalizations in the United States. Obstet Gynecol. 2012;120(5):1029–36. Epub 2012/10/24. http://10.1097/AOG.0b013e31826d60c5. . 23090519 10.1097/aog.0b013e31826d60c5

[2] Campbell KH, Savitz D, Werner EF, Pettker CM, Goffman D, Chazotte C, et al Maternal morbidity and risk of death at delivery hospitalization. Obstet Gynecol. 2013;122(3):627–33. Epub 2013/08/08. doi: 10.1097/AOG.0b013e3182a06f4e . 23921870 10.1097/AOG.0b013e3182a06f4e

[3] Callaghan WM, Mackay AP, Berg CJ. Identification of severe maternal morbidity during delivery hospitalizations, United States, 1991–2003. Am J Obstet Gynecol. 2008;199(2):133.e1–8. Epub 2008/02/19. doi: 10.1016/j.ajog.2007.12.020 . 18279820 10.1016/j.ajog.2007.12.020

[4] Torio C, Moore B. National Inpatient Hospital Costs: The Most Expensive Conditions by Payer, 2013. http://www.hcup-us.ahrq.gov/reports/statbriefs/sb204-Most-Expensive-Hospital-Conditions.pdf Accessed June 21, 2016: Healthcare Cost and Utilization Project, Agency for Healthcare Research and Quality, Rockville, MD; May 2016. 27359025

[5] Lazariu V, Kacica M. New York State Maternal Mortality Review Report, 2006–2008. https://www.health.ny.gov/community/adults/women/docs/maternal_mortality_review_2006-2008.pdf Accessed February 22, 2016.: New York State Department of Health, Division of Family Health, 2016.

[6] Gray KE, Wallace ER, Nelson KR, Reed SD, Schiff MA. Population-based study of risk factors for severe maternal morbidity. Paediatr Perinat Epidemiol. 2012;26(6):506–14. Epub 2012/10/16. doi: 10.1111/ppe.12011 ; 23061686 10.1111/ppe.12011 PMC3498497

[7] Howell EA, Zeitlin J, Hebert PL, Balbierz A, Egorova N. Association between hospital-level obstetric quality indicators and maternal and neonatal morbidity. Jama. 2014;312(15):1531–41. Epub 2014/10/17. doi: 10.1001/jama.2014.13381 . 25321908 10.1001/jama.2014.13381 PMC4334152

[8] Creanga AA, Bateman BT, Kuklina EV, Callaghan WM. Racial and ethnic disparities in severe maternal morbidity: a multistate analysis, 2008–2010. Am J Obstet Gynecol. 2014;210(5):435.e1–8. Epub 2013/12/04. doi: 10.1016/j.ajog.2013.11.039 . 24295922 10.1016/j.ajog.2013.11.039

[9] Lyndon A, Lee HC, Gilbert WM, Gould JB, Lee KA. Maternal morbidity during childbirth hospitalization in California. J Matern Fetal Neonatal Med. 2012;25(12):2529–35. Epub 2012/07/12. doi: 10.3109/14767058.2012.710280 ; 22779781 10.3109/14767058.2012.710280 PMC3642201

[10] Geller SE, Rosenberg D, Cox S, Brown M, Simonson L, Kilpatrick S. A scoring system identified near-miss maternal morbidity during pregnancy. J Clin Epidemiol. 2004;57(7):716–20. Epub 2004/09/11. doi: 10.1016/j.jclinepi.2004.01.003 . 15358399 10.1016/j.jclinepi.2004.01.003

[11] Bateman BT, Mhyre JM, Hernandez-Diaz S, Huybrechts KF, Fischer MA, Creanga AA, et al Development of a comorbidity index for use in obstetric patients. Obstet Gynecol. 2013;122(5):957–65. Epub 2013/10/10. doi: 10.1097/AOG.0b013e3182a603bb ; 24104771 10.1097/AOG.0b013e3182a603bb PMC3829199

[12] Zhang J, Meikle S, Trumble A. Severe maternal morbidity associated with hypertensive disorders in pregnancy in the United States. Hypertens Pregnancy. 2003;22(2):203–12. Epub 2003/08/12. doi: 10.1081/PRG-120021066 . 12909005 10.1081/PRG-120021066

[13] Marr L, Lennox C, McFadyen AK. Quantifying severe maternal morbidity in Scotland: a continuous audit since 2003. Current Opinion in Anesthesiology. 2014;27(3):275–81. doi: 10.1097/ACO.0000000000000079 24739249 10.1097/ACO.0000000000000079

[14] Main EK, Abreo A, McNulty J, Gilbert W, McNally C, Poeltler D, et al Measuring severe maternal morbidity: validation of potential measures. Am J Obstet Gynecol. 2016;214(5):643.e1–.e10. Epub 2015/11/20. doi: 10.1016/j.ajog.2015.11.004 . 26582168 10.1016/j.ajog.2015.11.004

[15] Kuklina EV, Whiteman MK, Hillis SD, Jamieson DJ, Meikle SF, Posner SF, et al An enhanced method for identifying obstetric deliveries: implications for estimating maternal morbidity. Matern Child Health J. 2008;12(4):469–77. Epub 2007/08/11. doi: 10.1007/s10995-007-0256-6 . 17690963 10.1007/s10995-007-0256-6

[16] Reichenheim ME, Zylbersztajn F, Moraes CL, Lobato G. Severe acute obstetric morbidity (near-miss): a review of the relative use of its diagnostic indicators. Arch Gynecol Obstet. 2009;280(3):337–43. Epub 2008/12/30. doi: 10.1007/s00404-008-0891-1 . 19112576 10.1007/s00404-008-0891-1

[17] Weight Gain During Pregnancy: Reexamining the Guidelines Institute of Medicine and National Research Council. 2009: Washington, DC: The National Academies Press; 2009. 20669500

[18] Alexander GR, Kotelchuck M. Quantifying the adequacy of prenatal care: a comparison of indices. Public Health Rep. 1996;111(5):408–18; discussion 19. Epub 1996/09/01. ; 8837629 PMC1381783

[19] New York State Department of Health. Perinatal Regionalization. https://www.health.ny.gov/community/pregnancy/health_care/perinatal/regionalization_descrip.htm Accessed February 3, 2016.

[20] Hardin J, Hilbe J. Chapman & Hall; Boca Raton: 2003. Generalized Estimating Equations.

[21] Survey Methodology Program SRC, Institute for Social Research, University of Michigan. IVEware: Imputation and Variance Estimation Software.

[22] Raghunathan TE, Lepkowski JM, Van Hoewyk J, Solenberger P. A Multivariate Technique for Multiply Imputing Missing Values Using a Sequence of Regression Models. Survey Methodology2001. p. 85–95.

[23] Declercq E, Barger M, Cabral HJ, Evans SR, Kotelchuck M, Simon C, et al Maternal outcomes associated with planned primary cesarean births compared with planned vaginal births. Obstet Gynecol. 2007;109(3):669–77. Epub 2007/03/03. doi: 10.1097/01.AOG.0000255668.20639.40 . 17329519 10.1097/01.AOG.0000255668.20639.40

[24] Belanoff C, Declercq ER, Diop H, Gopal D, Kotelchuck M, Luke B, et al Severe Maternal Morbidity and the Use of Assisted Reproductive Technology in Massachusetts. Obstet Gynecol. 2016;127(3):527–34. Epub 2016/02/09. doi: 10.1097/AOG.0000000000001292 ; 26855105 10.1097/AOG.0000000000001292 PMC4764424

[25] Barrett M, Smith M, Elixhauser A, Honigman L, Pines J. Utilization of Intensive Care Services, 2011. http://www.hcup-us.ahrq.gov/reports/statbriefs/sb185-Hospital-Intensive-Care-Units-2011.pdf Accessed February 3, 2016: Healthcare Cost and Utilization Project, Agency for Healthcare Research and Quality; December 2014. 25654157

[26] Cantwell R, Clutton-Brock T, Cooper G, Dawson A, Drife J, Garrod D, et al Saving Mothers' Lives: Reviewing maternal deaths to make motherhood safer: 2006–2008. The Eighth Report of the Confidential Enquiries into Maternal Deaths in the United Kingdom. Bjog. 2011;118 Suppl 1:1–203. Epub 2011/03/05. doi: 10.1111/j.1471-0528.2010.02847.x . 21356004 10.1111/j.1471-0528.2010.02847.x

[27] Louis JM, Menard MK, Gee RE. Racial and ethnic disparities in maternal morbidity and mortality. Obstet Gynecol. 2015;125(3):690–4. Epub 2015/03/03. doi: 10.1097/AOG.0000000000000704 . 25730234 10.1097/AOG.0000000000000704

[28] Garland A, Yogendran M, Olafson K, Scales DC, McGowan KL, Fransoo R. The accuracy of administrative data for identifying the presence and timing of admission to intensive care units in a Canadian province. Med Care. 2012;50(3):e1–6. Epub 2012/01/25. doi: 10.1097/MLR.0b013e318245a754 . 22270100 10.1097/MLR.0b013e318245a754

[29] Blichert-Hansen L, Nielsson MS, Nielsen RB, Christiansen CF, Norgaard M. Validity of the coding for intensive care admission, mechanical ventilation, and acute dialysis in the Danish National Patient Registry: a short report. Clin Epidemiol. 2013;5:9–12. Epub 2013/01/30. doi: 10.2147/CLEP.S37763 ; 23359787 10.2147/CLEP.S37763 PMC3555432

[30] Yasmeen S, Romano PS, Schembri ME, Keyzer JM, Gilbert WM. Accuracy of obstetric diagnoses and procedures in hospital discharge data. Am J Obstet Gynecol. 2006;194(4):992–1001. Epub 2006/04/04. doi: 10.1016/j.ajog.2005.08.058 . 16580288 10.1016/j.ajog.2005.08.058

[31] Bouvier-Colle MH, Mohangoo AD, Gissler M, Novak-Antolic Z, Vutuc C, Szamotulska K, et al What about the mothers? An analysis of maternal mortality and morbidity in perinatal health surveillance systems in Europe. BJOG: an international journal of obstetrics and gynaecology. 2012;119(7):880–9; discussion 90. doi: 10.1111/j.1471-0528.2012.03330.x ; 22571748 10.1111/j.1471-0528.2012.03330.x PMC3472023

