# Supplementary material for: Severe maternal morbidity: A population-based study of an expanded measure and associated factors
Source: PLoS One. 2017 Aug 7;12(8):e0182343. doi: 10.1371/journal.pone.0182343 (PMC5546569; doi:10.1371/journal.pone.0182343)
Supplement: S1 Document — (PDF) [file pone.0182343.s001.pdf]

2014

# SPARCS

# Data Governance Policy and Procedure Manual

Bureau of Health Informatics
Office of Quality and Patient Safety
NYS Department of Health

Version 1.0 (Sep. 2014)

# Statewide Planning and Research Cooperative System (SPARCS)
# Data Governance Policy and Procedure Manual

## TABLE OF CONTENTS


Introduction ........ 2
Data Governance Committee Membership ........ 2
SPARCS Data Access ........ 2
SPARCS Data File Types ........ 3
- Identifiable Data ........ 3
- Limited Data ........ 4
- De-identified (Public Use Files) ........ 5

Identifiable Data: Request Process ........ 5
- Years of Data ........ 6
- Abortion and HIV/AIDS Data Requests ........ 6
- Linking Data Requests ........ 6

SPARCS Data Governance Committee Meetings ........ 6
- Pre-Meeting Activities ........ 6
- Pre-Meeting Checklist ........ 7
- During the Meeting ........ 7
- Post-Meeting Activities ........ 7
- Post-Ratification Checklist ........ 8

Previously Approved Data Request Actions ........ 8
- Amended Applications ........ 8
- Streamlined Applications ........ 8

Administrative Actions ........ 8
- Minor Project Changes ........ 9
- Changes in Project Director Requests ........ 9
- Extension of Data Retention Requests ........ 9
- Penalties ........ 9

SPARCS Data Retention ........ 9
Data Fees ........ 9
Contact Information ........ 10
Glossary of Terms ........ 11
Attachment A: Review and Recommendations Document ........ 13

## INTRODUCTION

The Statewide Planning and Research Cooperative System (SPARCS) is a comprehensive all payer data reporting system established in 1979 as a result of cooperation between the healthcare industry and government. The system was initially created to collect information on discharges from hospitals. SPARCS currently collects patient level detail on patient characteristics, diagnoses and treatments, services, and charges for inpatient and outpatient (ambulatory surgery, emergency department, and outpatient services), hospital services and outpatient services from free-standing ambulatory surgery centers.

The enabling legislation for SPARCS is located under Section 28.16 of the Public Health Law (PHL). The regulations pertaining to SPARCS are under Section 400.18 of Title 10 (Health) of the Official Compilation of Codes, Rules, and Regulations of the State of New York (NYCRR).

The SPARCS Data Governance Committee was formed in September 2014 to review identifiable data releases. The SPARCS Data Governance Committee supersedes the SPARCS Data Protection Review Board (DPRB). All previously approved DPRB requests are grandfathered in under the new governance structure.

SPARCS Operations is responsible for ensuring the quality, consistency, usability, security, and availability of all data requests from internal and external stakeholders seeking to use SPARCS data. SPARCS Operations follows all applicable federal and state laws when determining whether SPARCS data that contain identifiable data elements may be shared and whether a disclosure of SPARCS constitutes an unwarranted invasion of personal privacy.

SPARCS Operations supports open government initiatives and transparency while continuing to assure confidentiality and security of the patient data it is charged with protecting.

SPARCS Operations will publish and make publicly available the name of the project director, the organization, and the title of all approved projects that use identifiable data.

Monthly updates on SPARCS, including data release, is provided by the SPARCS Update newsletter available on the public website at http://www.health.ny.gov/statistics/sparcs/newsletters/.

## DATA GOVERNANCE COMMITTEE MEMBERSHIP

Membership on the SPARCS Data Governance Committee is a New York State Department of Health (NYSDOH) administered process. The Committee is comprised of at least three members, including at least one member not otherwise affiliated with the NYSDOH. Committee members will alternate between 2 and 3 year terms.

All committee members are identified by a SPARCS Administrator and confirmed by the Commissioner of Health.

The names and titles of current SPARCS Data Governance Committee members is made available on the SPARCS web page on the NYS DOH public web site at http://www.health.ny.gov/statistics/sparcs/.

## SPARCS DATA ACCESS

SPARCS data may be used for medical or scientific research or statistical or epidemiological purposes.

All entities seeking SPARCS identifiable or limited data must submit a request to SPARCS Operations using standard data request forms. Detailed information on the type of files available and the processes that are followed to approve use is contained within this manual.

Approved data users shall take all necessary precautions to prevent unwarranted invasions of personal privacy resulting from any data analysis or release. Data users may not release any information that could be used, alone or in combination with other reasonably available information, to identify an individual who is a subject of the information. Data users bear full responsibility for breaches or unauthorized disclosures of personal information resulting from use of SPARCS data. Applications for SPARCS data must provide an explicit plan for preventing breaches or unauthorized disclosures of personal information of any individual who is a subject of the information. Applications must also include a copy of the SPARCS Security Guidelines signed by the organizational representative.

Each data request form must include an executed data use agreement in a form prescribed by SPARCS Operations. Data use agreements are required of: a representative of the requesting organization; a representative of each other organization associated with the project; and all individuals who will have access to any data including identifiable data elements.

In determining the purpose of a request for SPARCS data, SPARCS Operations shall not be limited to information contained in the data request form and may request supplemental information from the applicant.

The Commissioner may rescind for cause, at any time, approval of a data request.

## SPARCS DATA FILE TYPES

There are three types of SPARCS data available to researchers and others wishing to use the data:

1. Identifiable
2. Limited
3. De-identified (Public Use)

The Data Governance Committee is responsible for the review and approval of **identifiable** data requests. Limited data request applications are reviewed and processed as part of SPARCS Operations. De-identified data is openly available to everyone. This section will detail the use of these data file types and the appropriate processes and procedures to obtain the data.

### IDENTIFIABLE DATA

Identifiable data pertains to a particular individual's facility stay which, if disclosed, would constitute an unwarranted invasion of personal privacy, as stipulated in Department regulations, Title 10, NYCRR 400.18.

The following documents are associated with requesting SPARCS Identifiable data, all of which can be downloaded from the NYS DOH public website at the following direct link: http://www.health.ny.gov/statistics/sparcs/forms/

**Identifiable Data Request Forms**

- Identifiable Data Overview and Instructions (DOH-4385 Instructions)
- Identifiable Data Request Form (DOH-4385 Application)
- Organizational Affidavit (DOH-4385OA)
  This form must be signed by a representative that has the authority to legally bind the organization.
- Individual Affidavit (DOH-4385IA)
  This form must be signed by the Project Director and every person that will use SPARCS data.

Permission to use SPARCS identifiable data can be obtained by completing an identifiable Data Request application (DOH-4385), and submitting it by e-mail to sparcs.requests@health.ny.gov.

Applications go through a preliminary review process by SPARCS Operations and changes are made (if needed). When an application is ready, it is presented to the SPARCS Data Governance Committee who will then vote on whether or not to approve the application. The Committee’s recommendations are sent to the Commissioner for ratification. The Commissioner’s decision is returned to the SPARCS Administrator within 10 business days. If approved, an invoice for the data will be sent (if applicable) and, upon receipt of payment, the data will be mailed. If the application is denied, a notification will be sent with the denial reason.

Below is the list of current identifiable element categories that can be requested and the SPARCS data elements they contain. Not all elements are available on all years of data. The flow chart presented to the left provides the process that is followed for SPARCS identifiable data requests.

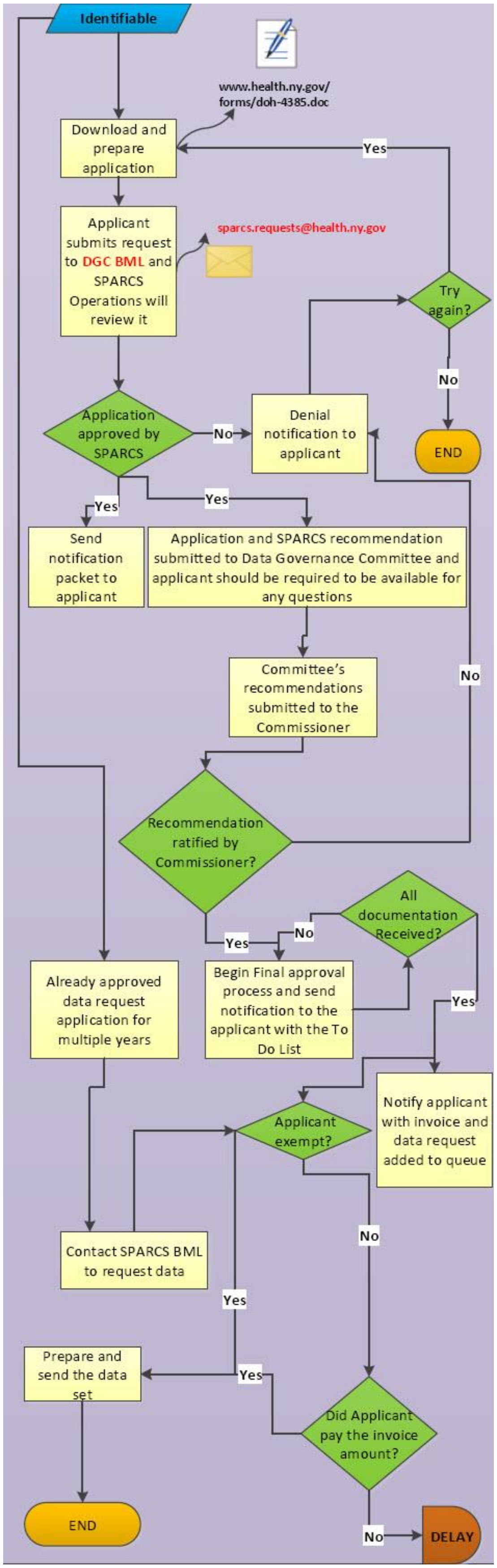


| Category | SPARCS Identifiable Data Element |
|---|---|
| **Dates** | Accident Related Date |
| | Admission Date |
| | Date Alternate Care Required |
| | Date of Service |
| | Date of Visit |
| | Discharge Date |
| | Non-Acute Care From Date |
| | Non-Acute Care Thru Date |
| | Principal Procedure Date |
| | Other Procedures 1-4, 5, 6-14 |
| | Statement Covers From Date |
| | Statement Covers Thru Date |
| **Date of Birth** | Patient Date of Birth |
| **Address** | Residence Address Line 1 |
| | Residence Address Line 2 |
| | Patient Zip Code Extension |
| **Numbers** | Medical Record Number |
| | Mothers Medical Record Number (for newborn) |
| | Patient Control Number |
| | Pre-Hospital Care Report Number |
| **Policy Number** | Policy Number |
| **UPI** | Unique Personal Identifier |

## LIMITED DATA

Limited data contains indirect identifiers that are deemed potentially identifiable according to HIPAA standards. Access to the limited data file requires that we have a Data Use Agreement on file prior to use. A complete list of elements can be found in the output data dictionary (inpatient or outpatient), located on the public webpage at http://www.health.ny.gov/statistics/sparcs/datadic.htm.

The process to request the use of limited data can be started by reviewing the instructions and completing the limited Data Request Form found on the public website at http://www.health.ny.gov/statistics/sparcs/forms/.

Limited Data Request Forms

- Non-Identifiable Data Overview and Instructions (DOH-4395 Instructions)
- Non-Identifiable Data Request Form (DOH-4395)

When complete, the application (including Data Use Agreement), should be e-mailed to sparcs.requests@health.ny.gov. Limited data applications are reviewed by SPARCS Operations staff and do not need to go to the Data Governance Committee for approval and the Commissioner for ratification. If accepted, an invoice will be sent (if applicable), and upon receipt of payment the data will be mailed. If there is something wrong with the application, it will be returned to the applicant with instructions for correction.

## DE-IDENTIFIED (PUBLIC USE FILES)

Public use data files contain de-identified data consisting of basic record-level detail. Public use files do not contain protected health information (PHI) under HIPAA. Public use data is openly available at https://health.data.ny.gov/. By keying in "SPARCS" in the search box, users are presented with a list of available data, by year.

Also available to the public is a Health Data Query System at the following direct link: https://apps.health.ny.gov/pubdoh/sparcsqry/. SPARCS discharge data is summarized in statistical tables on an annual calendar year basis. These tables are grouped into the following major areas: Statewide, County, and Hospital. Some of the specific data categories included in these tables are: Age, Gender, and Expected Principal Source of Reimbursement, Service Category, Major Diagnostic Category, and Disposition of Patient.

## IDENTIFIABLE DATA: REQUEST PROCESS

The DOH-4385 is the application submitted by the requestor indicating: data year(s) and elements requested, request purpose, individual(s) that will access the data, and storage security compliance. Identifiable data requests are granted only upon formal, written approval for access by a majority of the members of the Committee. The Commissioner has the final authority over the approval, or disapproval, of all requests.

All applications are subjected to a thorough administrative review resulting in a Review and Recommendation (R&R) document (Attachment A), which is created to assist Committee members with their assessment. It summarizes the application request and includes comments by the reviewers. The following items are verified during the R&R process:

- the type of project,
  - Epidemiological
  - Financial
  - Health Planning and Resource Allocation
  - Quality of Care Assessment
  - Rate Setting
  - Research Studies
  - Surveillance
  - Utilization Review of Resources
- the feasibility and soundness of the project,
- the research methodology,
- the security configuration protecting the data,
- IRB Approval, and
- dissemination and linkages protect the privacy of the patient.

Most applications require multiple phone interviews with the project director or coordinator to meet these requirements. Correspondence and related files are tracked both electronically and in paper format.

Requests for identifiable data elements shall be considered and/or approved by the Committee only if:

(i) The purpose of the request is consistent with the purposes for which SPARCS data may be used;

(ii) The applicant is qualified to undertake the project; and

(iii) The applicant requires such identifiable data elements for the intended project and is able to ensure that patient privacy will be protected.

## YEARS OF DATA

SPARCS Inpatient and Outpatient data are currently available for the following years:

- Inpatient (1982-present)
- Outpatient:
    - Ambulatory Surgery (1983-present)
    - Emergency Department (2005-present)
    - Expanded Outpatient Data Collection (EODC) (2011-present)

SPARCS data may be requested for a predetermined time not to exceed three years beyond the current year provided that the organization and uses of the data remain as indicated in the data request form submitted to SPARCS Operations.

## ABORTION AND HIV/AIDS DATA REQUESTS

Abortion and HIV/AIDS records are redacted to the standard of the HIPAA "Safe Harbor" provision, which eliminates all direct and indirect patient identifiers.

HIV/AIDS data release follows PHL § 2135. Redacted elements can be released to any researcher with patient consent, or to another public health entity if approved by the Committee. Any researcher can apply for, and if approved, receive the indirect identifiers (Safe Harbor).

Abortion data elements cannot be released to researchers, unless the request is accompanied by patient consent forms.

## LINKING DATA REQUESTS

Linking SPARCS data to other data sources are completed by the researcher, providing that the SPARCS Operations receives approval from the owners of the other data source(s). This is contingent upon proof that a qualified individual will perform the match and that any unused/un-needed data will be destroyed or returned to the Department.

# SPARCS DATA GOVERNANCE COMMITTEE MEETINGS

Convened by the SPARCS Administrator, Data Governance Committee meetings are held eight times a year. A series of eight (8) 2 hour meetings (conference calls) are scheduled (pre-set) over a 12 month period. These are the dates during which the committee will meet to discuss applications. Committee members are asked when the dates are set if they anticipate they will be able to attend and vote at the established time, but a quorum is not required to hold a meeting. Committee members are allowed to vote on applications even if they are not present at the meeting. Individuals who are not committee members may attend to listen and speak (but not vote) at committee meetings.

## PRE-MEETING ACTIVITIES

The application package and the R&R are distributed to members of the Data Governance Committee one week before the meeting. The applicant also receives a copy of the R&R and has an opportunity to clarify or revise information one final time before the meeting. The R&R also informs the applicant about missing documentation that may delay the request process.

Data Governance Committee members are provided with a full set of documentation materials related to the data requests to be reviewed at least a week before the scheduled vote on approval of the application. Members are required to review the request and bring any questions or concerns that were not answered prior to the meeting, to the meeting for discussion and group review.

## PRE-MEETING CHECKLIST

The following table is a list of steps prior to the Data Governance Committee meeting where the applicants request for SPARCS data will be discussed and then voted upon.

### TABLE I: PRE-MEETING CHECKLIST

| Tasks | Notes | Deadline |
|---|---|---|
| **Receipt of Application** | SPARCS Operations enters the application, and affidavits, into an electronic tracking system, assigns a tracking number to the application, makes a photocopy for the Committee reviewers, creates a hard copy folder, and sends "application received" notification to applicant. | No later than 2 weeks prior to meeting |
| **Scan Applications** | Applications presented to the Committee for approval must be scanned and saved in meeting folder. | Within 1 week of meeting |
| **Preliminary Review** | The application is reviewed for accuracy and completeness. Create R&R. | 2 weeks prior to meeting |
| **Prepare and Mail Pre-Meeting Packages** | For applicants: letter to applicant, copy of R&R, copy of their application.<br>For review members: applications, R&R, draft agenda. | 1 week prior to meeting |
| **Prepare Meeting Day Paperwork** | Sign-in sheets, voting sheets, applicant contact list. | 1 day before meeting |

## DURING THE MEETING

During the Data Governance Committee meeting, the applicant should be available by phone to respond to any follow up questions that are critical to the Committee vote on the application. If the applicant is unavailable the request can be deferred until the next meeting.

Committee members ensure that the applicant is qualified to conduct the research or study, has the appropriate credentials, meets security requirements, and has a just and sound project. They also ensure that the data elements being requested are required to meet the project goals.

Following review of the application, the applicant may be asked questions by the Committee members. The Committee then votes on the application and, by a majority ruling, whether it recommends approval or not.

## POST-MEETING ACTIVITIES

If approved by the Committee, and before the applicant can receive the data, the approved request is submitted to the Commissioner of Health for ratification. This process is completed within 10 business days of voting. If the Commissioner does not ratify the Committee's decision, the application is returned to the applicant. The applicant may apply again.

SPARCS Operations sends a notice to the applicant informing them of the decision and, if necessary, requesting any documentation that must be submitted before the data can be released.

Upon receipt of all documentation, the applicant will receive e-mailed instructions on how to obtain the data.

The request is then entered into the data request queue, where the approved dataset will be created. Upon receipt of payment (if due), the data will be shipped to the applicant. Data is typically released within 30 days of ratification.

### TABLE II: POST-MEETING CHECKLIST

| Tasks | Notes | Deadline |
|---|---|---|
| **Ratification Package** | E-mail Ratification Package. Includes: Commissioner signature page for each app.; SPARCS administrator signature page for each app.; and abridged R&R. | Within 1 business day |
| **Data Entry** | Data approved by the Committee must be entered into the electronic tracking system. | Within 1 week of meeting |

## POST-RATIFICATION CHECKLIST

### TABLE III: POST-RATIFICATION CHECKLIST

| Tasks | Notes | Deadline |
|---|---|---|
| **Decision Notification Letters** | Letters are e-mailed to the applicant along with a copy of the approved data elements, and a list of any outstanding documentation. | Within 1 business day of ratification |
| **Dataset sheets** | Dataset sheets are completed for each application. They contain all information required to cut the specific file. When complete, the final approval process can begin. | While waiting for ratification |
| **Final Approval Process** | Once all documentation is in and all conditions met, the file will be reviewed to make sure all affidavits are present and the data entered is correct. If all requirements are met, the request is date stamped with the final approval. | Within 1 business day of receipt of ratification package |
| **Final Approval Notification** | Notify the applicant of final approval instructions via e-mail. Upon receipt of the notification, applicants must contact sparcs.submissions@health.ny.gov to request the approved data. | Within 1 business day of final approval |

# PREVIOUSLY APPROVED DATA REQUEST ACTIONS

## AMENDED APPLICATIONS

Amended applications are supplied in cases where the requestor is making changes to a previously approved application. An amended application can be requested by e-mailing sparcs.requests@health.ny.gov. Once received, the completed application will go through the same process as an identifiable request.

## STREAMLINED APPLICATIONS

Streamlined applications are supplied in cases where the only change is the addition of years of data to a currently approved application. A streamlined application can be requested by e-mailing sparcs.requests@health.ny.gov. This type of request is also presented to the Committee, but the applicant is not required to be available during the meeting.

# ADMINISTRATIVE ACTIONS

Certain actions that meet specific guidelines can be approved administratively by the SPARCS Administrator, such as an extension of project completion, a change in Project Director, minor changes to the project scope, and analysis of additional elements. All requests must be submitted to sparcs.requests@health.ny.gov. All administrative requests will be mentioned at the next scheduled Committee meeting under the topic “Administrative Actions.”

## MINOR PROJECT CHANGES

Requests can be made to make minor changes, for example, the review of additional CPT codes (data already received), and to make minor changes to scope or goal of the project.

## CHANGES IN PROJECT DIRECTOR REQUESTS

A request to change the Project Director must be made by the original Project Director. Included in the e-mail request must be the name of the new Project Director, their Curriculum Vitae (CV), and their Individual Affidavit. If the original Project Director is no longer available, the request can come from the Organization Representative that co-signed the application.

## EXTENSION OF DATA RETENTION REQUESTS

In situations where more time may be required to complete the study, the data retention period may be extended for up to two years. All extension requests must be submitted to sparcs.requests@health.ny.gov for SPARCS Operations review.

## PENALTIES

Breaches or other infractions of the Data Use Agreement can result in penalties against the requestor, the organization, or both. These can include monetary fines, denial of any future use of SPARCS data, or rescinding the use of SPARCS data for the entire organization.

# SPARCS DATA RETENTION

The data retention period is two years after the final year of data has been received. The project is closed when retention expires, upon project completion and the return of the data, or upon withdrawal of the request. During the period of retention, no additional individuals may access SPARCS data without an approved individual data use agreement on file with SPARCS Operations.

# DATA FEES

SPARCS Operations charges a reasonable fee to all persons and organizations receiving SPARCS data based upon costs incurred and recurring for data processing, platform/data center and software. The Commissioner may discount the base fee or waive the fee upon request to the SPARCS program. The fee may be waived in the following circumstances:

(i) Used by a health care facility of the data it submitted to SPARCS.
(ii) Used by a health care facility that is licensed under Article 28 of the Public Health Law for the purpose of rate determinations or rate appeals and for health care-related research.
(iii) Used by a Federal, New York State, county or local government agency for health care-related purposes.

To obtain the current price for SPARCS identifiable and limited data sets, please e-mail sparcs.submissions@health.ny.gov.

# CONTACT INFORMATION

SPARCS Operations is located within the Bureau of Health Informatics (BHI) in the Office of Quality and Patient Safety (OQPS). Please contact us at:

SPARCS Operations
Bureau of Health Informatics
Office of Quality and Patient Safety
New York State Department of Health
Empire State Plaza
Corning Tower Room 1970
Albany, New York 12237

| | |
|---|---|
| Phone: | (518) 474-3189 |
| Fax: | (518) 486-3518 |
| E-mail: | sparcs.submissions@health.ny.gov (General Inquiries) |
| | sparcs.requests@health.ny.gov (Data Requests) |
| | bhi@health.ny.gov (All Other Inquiries) |
| ListServ: | sparcs-l@health.state.ny.us |
| Website: | http://www.health.ny.gov/statistics/sparcs/ |


SPARCS Operations has made every effort to provide accurate and complete information in this manual. Any typographical error is unintentional on our part and we urge users of this manual to bring them to our attention for correction. Edits, deletions, modifications, or changes to areas of this manual will be maintained in a change log and updated versions of the manual will be released.

## GLOSSARY OF TERMS

| Term | Description |
|---|---|
| **Affidavit: Individual** | The individual data use agreement that must be signed by the Project Director, and all individuals that will be working with SPARCS data. |
| **Affidavit: Organizational** | A binding document that is required to be signed by a representative of the organization who is authorized to sign on behalf of said organization. This person is usually a Corporate Officer, or Department Chair. |
| **Breach** | An unauthorized use of SPARCS data, either by a person without an individual affidavit on file, or by using the data in a manner, or for a purpose not approved by the Data Governance Committee. |
| **Data Governance Committee** | A group of people experienced with SPARCS data, and/or research and that will review the application for identifiable data, and make a recommendation to the Commissioner whether or not to allow the release of the data. Recommendations must be ratified by the Commissioner and the group's decisions can be overturned. |
| **Data Protection Review Board** | The previous Board that presided over the request and presentation of SPARCS data applications through June 2014. They ensured that SPARCS data was released only to researchers with a proven ability to maintain a secure environment for the data, and that they were able to effectively perform the research. |
| **Data Queue** | After being approved to use the data, the applicant is notified to contact SPARCS@health.ny.gov to request whichever year(s), and file types, they are ready to receive. At that point, they are placed in the queue with other data requesters, and their file will be cut when they reach to top of the queue. The length of one's wait in the queue varies on the number of requesters in the queue at that time, and the complexity of the data cut. A SPARCS Operations goal is to complete each request within 30 days. |
| **Data Retention** | The date at which each applicant is allowed to use SPARCS data. This date is set programmatically to two years after the last year of data is received. Retention can be extended for up to two additional years by e-mailing a request to sparcs.requests@health.state.ny.gov (include the reason for the extension). |
| **De-identified Data** | Unlike the identifiable data, this file does not contain data that can be used (alone, or linked to other data), to identify a patient. An inpatient public use file is located on www.health.data.ny.gov simply by searching for keyword "SPARCS" |
| **Health Data Query System** | This report tool is located on our public webpage at https://apps.health.ny.gov/pubdoh/sparcsqry/ and creates listings or comparisons by year, facility, county, HSA or CCA on patients, days and charges. |
| **Identifiable Data** | SPARCS data elements, which if used inappropriately, could identify a patient. This type of data is also referred to as Protected Health Information (PHI). All identifiable data requests are reviewed by the Data Governance Committee. |
| **IRB** | Institutional Review Board. |
| **Inpatient** | SPARCS currently collects patient level detail on characteristics, diagnoses and treatments, services, and charges for inpatient stays in New York State from 1982-present. |
| **Limited Data** | Limited data contains indirect identifiers that are deemed potentially identifiable according to HIPAA standards. |
| **NYCRR 400.18** | The section of the New York Codes, Rules and Regulations that allow SPARCS to collect data from facilities, and the manner in which they are allowed to release that data. |

| Term | Description |
| --- | --- |
| **NYSDOH** | The New York State Department of Health |
| **Outpatient** | SPARCS currently collects patient level detail on characteristics, diagnoses and treatments, services, and charges for outpatient visits in New York State for the following types:<br>• Ambulatory Surgery (1983-present)<br>• Emergency Department (2005-present)<br>• Expanded Outpatient Data Collection (EODC) (2011-present) |
| **Public Health Law (PHL), 2816** | The New York Public Health Law that defines SPARCS. |
| **Ratification** | The Commissioner of Health's decision on the Committee's recommendations to release SPARCS data to the requesting researcher. |
| **Review and Recommendation (R&R)** | The document created by SPARCS Operations while reviewing an applicant's request (Attachment A). It is used to assist the Committee with their recommendation. |
| **Safe Harbor** | HIPAA "Safe Harbor" is the de-Identification of Medical Record Information requiring that certain identifiers of the individual or of relatives, employers, or household members of the individual must be removed from medical record information in order for the records to be considered de- identified. |
| **SPARCS Administrator** | Person responsible (or his designee), for assuring that NYCRR Title 10 Section 400.18 is adhered to when requesting access to SPARCS identifiable data. |
| **SPARCS Operations** | The program area within the Bureau of Health Informatics, Office of Quality and Patient Safety that oversees all SPARCS program functions, including the collection and release of SPARCS data. |
| **Transparency** | The Department of Health is committed to creating a level of openness that builds trust and accountability. |
| **Data Set Sheet** | A document that is created upon finalizing an applicant's request. It contains the information necessary to create the specific data file. |

# ATTACHMENT A: REVIEW AND RECOMMENDATIONS DOCUMENT

| **New York State Department of Health**<br>**Bureau of Health Informatics**<br>**SPARCS Operations Memorandum** | **Agenda Item** |
|---|---|
| Applicant: | |
| Organization: | |
| Request Type: _____ New _____ Amended _____Streamlined | |
| Date: | |

**OVERVIEW**

Title: ________________________________________________________________________

________________________________________________________________________

| | |
|---|---|
| Previous Applications: _____Yes _____No | _____ HIV/AIDS _____ Data Linkages<br>_____Abortion _____ Secondary Release |

Data Requested:

| Type: | Years: |
|---|---|
| Inpatient | _____________________ |
| Outpatient | _____________________ |
| PRI | _____________________ |

1. If approved for HIV/AIDS, the address will include the 5-digit zip, county and regional elements only.
2. Data approved for release to the requestor cannot be secondarily released to any other person unless expressly approved.
3. We must be kept apprised of all publications, website postings, etc. derived from SPARCS data.
4. Statistical tabulation of facility records will have no single grouping or tabulation based upon fewer than six facility patient records. Such groupings will be eliminated or combined with other groupings.

**REVIEW**

Summary:

| Documentation Received (please check) | |
|---|---|
| | Organizational Affidavit(s) |
| | Individual Affidavit(s) |
| | Signature-project director |
| | Signature-organizational rep |
| | Qualifications |
| | Certification of Destruction Letter |
| | Security Requirements |
| | Vendor / contractor affidavits |
| | Vital Statistics Approvals |
| | Institutional Review Board (IRB) Approval |

| **Elements Recommended** | **Decision** |
|---|---|
| Dates | Y/N |
| DOB | Y/N |
| Address | Y/N |
| Numbers | Y/N |
| Policy Number | Y/N |
| Unique Person Identifier | Y/N |
