# Supplementary material for: Severe maternal morbidity: A population-based study of an expanded measure and associated factors
Source: PLoS One. 2017 Aug 7;12(8):e0182343. doi: 10.1371/journal.pone.0182343 (PMC5546569; doi:10.1371/journal.pone.0182343)
Supplement: S2 Document — (PDF) [file pone.0182343.s002.pdf]

Series: 500.0

Title: Access to Birth and Death Certificate Information

Date: 10/20/2014

POLICY

The use of birth and death certificate information by Department staff must be in compliance with existing laws, rules, regulations, protocols, and Department policies.

SCOPE

This item is applicable to all Department of Health offices and applies to identifying birth and death certificate data collected on birth and death certificates filed in New York State outside of New York City. Identifying data elements are consistent with HIPAA standards although the current identifying data element list may be more restrictive based on other statutory requirements. Current identifying data elements are listed in Attachment A.

INFORMATION

Birth and death certificates are an important source of public health data needed by the Department of Health to fulfill a wide variety of statutory and health program related functions. Data collected on birth and death certificates are used for statistical reporting, epidemiological analyses, program evaluation, and surveillance and in support of health program activities. Since birth and death certificates contain confidential and identifying information, great care is necessary to protect the personal privacy of the persons named on them.

While Article 41 of the Public Health Law specifically authorizes the use of death certificate information for research purposes, no specific provision for research use exists for birth certificate information including identifying data. However, Article 41 does authorize the use of birth certificate information when required for official purposes. Access to de-identified birth and death certificate data is available to Department programs for “proper purpose” (PHL 4174 1(e)). The following describes the process for considering and evaluating requests for access to identifying birth and death certificate information by Department programs.

Applicants must submit a **Request for Access to New York State Vital Records Data (DOH-4269)** to the Bureau of Vital Records (BVR) in the Division of Administration for review by BVR and the Bureau of Vital Statistics (BVS) in the Office of Quality and Patient Safety. Form DOH-4269 is available on the OQPS Intranet site (http://oqps/bor/vsu.html).

> ***Note - Access to New York City Birth and Death Data:*** *The New York State Department of Health is not authorized to provide access to New York City birth and death data. Under no circumstances will New York City birth or death data be released without the express written permission of the New York City Department of Health and Mental Hygiene.*

Access to identifying birth and death certificate information will be approved only for those projects where a well-defined public health need or benefit **directly** linked to the program’s mandate, mission or goals is clearly demonstrated, and, where risk of an invasion of personal privacy is outweighed by the benefits of the proposed project.

For review purposes, submitted projects will be classified as one of five categories:

> **Category 1 -** Non-research projects that involve identifying birth information, do not involve follow-back*, and are directly related to DOH program mandates, program evaluation, or surveillance.

*Follow-back is defined as contact with the individual, the individual's family, or any organization that would contact directly the individual or the family concerning the vital event.

Category 2 - Non-research projects that involve identifying death certificate information, with or without follow-back, and are directly related to DOH program mandates, program evaluation or surveillance.

Category 3 - Non-research projects that involve identifying birth certificate information, with follow-back, and are directly related to DOH program mandates, program evaluation or surveillance.

Category 4 -Research projects that involve identifying death certificate information, with or without follow-back, related to DOH program mandates, program evaluation or surveillance.

Category 5 -Research projects that involve identifying birth certificate information, with or without follow-back, that are related to DOH program mandates, program evaluation or surveillance.

Programs requesting use of identifying birth and death certificate information must obtain the endorsement of a Division Director or higher level manager. Among other requirements, the application for access to birth or death data must address the following:

- How the project relates to DOH program mandates, mission or goals
- The need for identifying information
- Other sources of identifying information that were considered and why they are deemed unsuitable
- Measures taken to safeguard confidentiality
- The need for follow-back, if applicable, and how it will be accomplished
- The need for case controls, if applicable, and why other sources are unsuitable
- Any plan for secondary release
- Any plan to publish results
- Any plan to link to other identified data sets

Applicant Information

The Project Director and Division Director must complete and sign the application.

The Division Director must sign a statement affirming that the project relates to the fulfillment of DOH mandates, mission or goals. The Project Director and all staff involved in the project will be required to sign a Data Use Agreement.

Some program areas may require approval at the program, Division or Center level of requests submitted to the Institutional Review Board. Therefore, the Project Director must secure and present documentation of such approval with the application (DOH-4269).

When completed, the application is submitted to the BVR for preliminary review with the BVS.

Bureau of Vital Records (BVR) / Bureau of Vital Statistics (BVS) Review

BVR and BVS will:

- Classify the project, i.e., Category 1, 2, etc.
- Review the application for completeness and content
- Determine if the request for identifying information can be met instead through BVS analysis, with use of a de-identified data set or through the use of proxy identifiers
- Discuss issues and alternatives with Project Director
- Submits Category 3, 4 and 5 to Institutional Review Board

If the requested use of identifying birth or death certificate data is classified as a Category 1 request, **Non-research project that involves identifying birth certificate information, does not involve follow-back, and is directly related to DOH program mandates, program evaluation, or surveillance** or a Category 2 request, **Non-research project that involves identifying death certificate information, with or without follow-back, and is directly related to DOH program mandates, program evaluation or surveillance**, the data may be made available to the program with no review by the Institutional Review Board or Executive Deputy Commissioner. For example, birth data when needed for quality assurance purposes involving a statutorily mandated registry may be approved by BVR/BVS, **provided that follow-back is not involved**.

If the requested use of the birth data is classified as a Category 3, 4, or 5 request, BVR/BVS will submit the request with recommendations to the Department's Institutional Review Board.

Institutional Review Board (IRB) Review

The IRB will evaluate the application to determine the appropriateness and scientific merit of the project/ research.

<u>Requests involving identifying birth certificate information (Categories 3 or 5)</u> - If the appropriateness and scientific merit of the project/ research are approved, the IRB will submit the application for Executive Deputy Commissioner consideration. If the appropriateness and scientific merit of the project/ research are not approved, the IRB may discuss the issues with the Project Director. If such issues are not satisfactorily resolved, the IRB will send a disapproval letter to the Project Director.

<u>Requests involving identifying death certificate information (Category 4)</u> - If the appropriateness and scientific merit of the project/ research is approved, the IRB will send the Project Director an approval letter. If the appropriateness and scientific merit of the project/ research is not approved, the IRB may discuss the issues with the Project Director. If such issues are not satisfactorily resolved, the IRB will send a disapproval letter to the Project Director.

Executive Deputy Commissioner Review

The Executive Deputy Commissioner will review all research and/or follow-back projects involving identifying birth certificate information to determine that:

1. the project is related to DOH program, goals and mission objectives,
2. any follow-back component of the project is appropriate,
3. the benefits of using identifying birth certificate information outweigh the risks of a possible unwarranted invasion of personal privacy.

Note: *Program or research use of identifying death data does <u>not</u> require Executive Deputy Commissioner approval.*

Upon request, the DLA will provide consultation to the Executive Deputy Commissioner.
The request for access to Vital Records data is returned to the IRB with the Executive Deputy Commissioner's determination. The IRB notifies the Project Director of approval or disapproval.

PROCEDURE

Department Applicant – Project Director	1.	Completes “Request for Access to New York State Vital Records Data” (DOH-4269) application form and submits to Division Director.<br><br>Some program areas may require approval at the program, Division or Center level of requests submitted to the Institutional Review Board. Therefore, the Project Director must secure and present documentation of such approval with the application (DOH-4269).
Division Director or Higher Level Manager in applicant's chain of command	2.	Reviews project and signs affidavit attesting to relationship of project to program’s mandate, mission or goals.
Project Director	3.	Submits completed application to BVR.
Bureaus of Vital Records (BVR) and Vital Statistics (BVS)	4.	Reviews application.<br><br>Classifies the project, i.e., Category 1, 2, etc.<br><br>Reviews the application for completeness and content.<br><br>Determines if the request for identifying information can be met instead through BVS analysis, with use of a de-identified data set or through the use of proxy identifiers.<br><br>Discusses issues and alternatives with Project Director
	5.	Classifies project as Category 1 (birth data, non-research, no follow-back), Category 2 (death data, non-research with or without follow back), Category 3 (birth data, non-research with follow-back), Category 4 (death data, research with or without follow-back), or Category 5 (birth data, research with or without follow-back).<br><br>If project is classified Category 1 or 2, **GO TO STEP 6**<br><br>If project classified as Category 3, 4, or 5, refers to IRB. **GO TO STEP 7**
	6.	If approved, sends approval letter to Project Director.<br><br>**GO TO STEP 10**<br><br>If not approved, sends disapproval letter to applicant, identifying deficiencies and providing opportunity to discuss and resubmit.<br><br>**STOP**
IRB	7.	Assesses the appropriateness and scientific merit of the project/research.<br><br>Category 3 or 5 Requests –<br><br>• If not approved, the IRB may discuss the issues with the Project Director.

- If such issues are not satisfactorily resolved, the IRB will send a disapproval letter to the Project Director.
- If approved, the IRB will submit for Executive Deputy Commissioner consideration.

Deputy Executive Commissioner or Designee	8.	Determines that project/research:<br>• relates to DOH program goals and mission<br>• any follow-back component is appropriate<br>• benefits of using birth certificate information outweigh the risks of a possible invasion of privacy<br><br>Seeks consultation of the Division of Legal Affairs, IRB, BVR, BVS, and Project Director as needed.<br><br>Returns decision to approve or disapprove project to the IRB
IRB	9.	If approved by Deputy Executive Commissioner or Designee, sends approval letter to applicant and BVR<br><br>If not approved by Deputy Executive Commissioner or Designee, IRB sends disapproval letter to applicant and BVR.
Applicant	10.	If project approved, works with BVR/BVS to obtain access to the data.
