# Supplementary material for: Severe maternal morbidity: A population-based study of an expanded measure and associated factors
Source: PLoS One. 2017 Aug 7;12(8):e0182343. doi: 10.1371/journal.pone.0182343 (PMC5546569; doi:10.1371/journal.pone.0182343)
Supplement: S3 Document — (DOC) [file pone.0182343.s003.doc]

NEW YORK STATE DEPARTMENT OF HEALTH **Request for Access to New York State Vital Records Data**

Bureau of Production Systems Management

| Send application to:  Director’s Office  Bureau of Production Systems Management  New York State Department of Health  P.O. Box 2602  Albany New York 12220-2602 | **For Office Use Only:** |
| --- | --- |
|  |  |
|  | Request # ____________________________ |
|  |  |
|  | Date Received ________________________ |
|  |  |
|  | Date Reviewed ________________________ |
|  |  |
|  | Approved: Yes ⬜ No ⬜  Conditions of Approval: |

## **APPLICANT ORGANIZATION INFORMATION**

| Agency: |  |
| --- | --- |

| Office/Bureau/Section: |  |
| --- | --- |

| Project Director: | Name: |  |
| --- | --- | --- |

|  | Title: |  |
| --- | --- | --- |

|  | Address: |  |
| --- | --- | --- |

|  |  |  |
| --- | --- | --- |

|  |  |  |
| --- | --- | --- |

|  | Telephone: |  |  |
| --- | --- | --- | --- |

|  | Email: |  |
| --- | --- | --- |

| Applicant Signature⏵ |  | Date: |  |
| --- | --- | --- | --- |

Supervisor Approval: *(If access is being requested by a New York State Department of Health program, the approving supervisor must be a Division Director level manager or higher.)*

I have read and approve this application for the data and purpose specified and I affirm that this project is directly related to the program’s mandate, mission or goals.

| Name: |  | Title: |  |
| --- | --- | --- | --- |

| Signature⏵ |  | Date: |  |
| --- | --- | --- | --- |

***Attach as many pages as necessary to completely answer each item.***

## **NATURE OF REQUEST**

| Project Title: |  |
| --- | --- |

### Is identifying information requested (See list of items considered identifying or potentially identifying in Attachment A)?

No  Yes  *If the answer is* Yes, *your application will be submitted for review by the Department of Health Institutional Review Board (IRB). Please contact the Bureau of Production Systems Management for further information*.

### Specify program name and section of Public Health Law establishing mandate.

*For example, Cancer Registry, Public Health Law Section 2401(8).*

| Program Name: |  |
| --- | --- |

| Public Health Law / NYCRR: |  |
| --- | --- |

### Describe in detail how the project is directly related to the program’s mandate, mission or goals:

|  |
| --- |

### Specify type of Vital Records file(s), i.e., live birth, death, and year(s) or time period(s) for which access is requested:

| |  | **Type of File** |  | **Year(s)** |  | | **Type of File** | |  | | **Year(s)** | | | --- | --- | --- | --- | --- | --- | --- | --- | --- | --- | --- | --- | | 1) |  |  |  | 2) |  | |  | |  | |  | | 3) |  |  |  | 4) |  | |  | |  | |  | | 5) |  |  |  | 6) |  | |  | |  | |  | |
| --- | --- | --- | --- | --- | --- | --- | --- | --- | --- | --- | --- | --- | --- | --- | --- | --- | --- | --- | --- | --- | --- | --- | --- | --- | --- | --- | --- | --- | --- | --- | --- | --- | --- | --- | --- | --- | --- | --- | --- | --- | --- | --- | --- | --- | --- | --- | --- | --- |

|  | **Type of File** |  | **Year(s)** |  | | **Type of File** |  | | **Year(s)** | | |
| --- | --- | --- | --- | --- | --- | --- | --- | --- | --- | --- | --- |
| 7) |  |  |  | 8) |  | | |  | |  |  |
| 9) |  |  |  | 10) |  | | |  | |  |  |
| 11) |  |  |  | 12) |  | | |  | |  |  |

- - 1. List identifying data elements to be provided to Vital Records in order to match requested records (e.g., name, date of birth, date of death, social security number, address, ZIP code, etc.):

|  |  |  |  |  |
| --- | --- | --- | --- | --- |
|  |  |  |  |  |
|  |  |  |  |  |
|  |  |  |  |  |

| - - 1. Estimate the number of records to be requested: |  |
| --- | --- |

- - 1. Computer File Abstracts:

Yes  Will accept computer file abstracts. *Go to F. below.*

No  Require certificate copies. *Please explain why certificate copies are required.*

*Explain:*

|  |
| --- |

### Is access to New York City records requested?

No  Yes  *If access to New York City Vital Records is requested, the express written consent of the New York City Department of Health is required.*

*In this case, please contact:*

Director, Quality Assurance and Special Studies

Bureau of Vital Statistics

New York City Department of Health

and Mental Hygiene

125 Worth Street, Box 7

New York, New York 10013

Telephone: 212-788-4585

### Describe the scope and intended use of the Vital Records data *(attach additional pages if necessary).*

### Include the following information:

1. Primary focus, including specific health, medical, statistical, quality assurance, legal or administrative issues that you plan to address;
2. Types of analyses to be performed;
3. Linkage with other data files, specifying type and source;
4. Benefits of the request;
5. Contact with the individual, next-of-kin, physicians, and/or other individuals or institutions mentioned on the Vital Records. Describe who will be contacted; how they will be contacted; information to be obtained from those contacted; methods to be used in obtaining information from those contacted; other organizations or consultants, if any, involved in the individual contacts.

|  |
| --- |

## **PROJECT ADMINISTRATION**

### Sponsors:

1. Are there any sponsors for this project?

No  Yes  *If the answer is* Yes, *give the name and address for each sponsor:*

|  |  |  |
| --- | --- | --- |
|  |  |  |
|  |  |  |
|  |  |  |
|  |  |  |

2. Will you be contracting with any organization to collect, process or analyze the Vital Records information?

No  Will **not** be contracting out. *Go to B. below.*

Yes  Will be contracting out. *Please provide contact information and describe the nature of the work to be performed by contractor:*

| Name of official: |  |
| --- | --- |
| Name of organization: |  |
| Address of organization: |  |
|  |  |
|  |  |

Work to be performed:

|  |
| --- |

###

### Specify all sources of funding for this project:

|  |
| --- |

### Protection of Human Subjects:

Has this project been reviewed and approved by an Institutional Review Board for the Protection of Human Subjects?

Yes  Give the name of the Review Board and the date of the approval*.*

**ATTACH A COPY OF THE APPROVAL TO THIS APPLICATION.**

| Name: |  |
| --- | --- |

| Date: |  |
| --- | --- |

No  *Indicate reasons*:

|  |
| --- |

### Informed Consent:

Have you or do you intend to obtain the written informed consent of the persons (individual, next-of-kin, legal representative) in your study to obtain copies of, or information from, their records?

Yes  **ATTACH A SAMPLE COPY OF THE CONSENT FORM TO THIS APPLICATION.**

No  *Indicate reasons*:

|  |
| --- |

## **CONFIDENTIALITY OF IDENTIFIABLE DATA**

**NOTE: Identifying data refers to any information that would permit, directly or indirectly, the identification of any individual or establishment.**

### A list of items considered identifying or potentially identifying information appears in Attachment A. Will any of these items be required by the project?

No  No identifying or potentially identifying information is required. *Go to B. below.*

Yes  *List below each of the identifying or potentially identifying items requested and the reason why each item is required:*

| **Identifying Data Element** |  | **Reason Requested** |
| --- | --- | --- |
|  |  |  |
|  |  |  |
|  |  |  |
|  |  |  |
|  |  |  |
|  |  |  |
|  |  |  |
|  |  |  |

### Will the requested data be linked to other data files?

No  The requested data will not be linked to data from other files. *Go to C. below.*

Yes  *List the file or files to which requested data may be linked* and

**ATTACH A LIST OF THE DATA ELEMENTS CONTAINED IN THE FILE(S).**

|  |  |  |
| --- | --- | --- |
|  |  |  |
|  |  |  |
|  |  |  |

### How will the confidentiality of any identifying data requested be maintained? (See NOTE above regarding identifying data.) Include an explanation of how such data will be secured as well as how and when you plan to dispose of the data after your project has been completed:

|  |
| --- |

## **SECONDARY RELEASE OF DATA**

### Will any data, identifying or non-identifying, from the requested Vital Records be released to other organizations (e.g. other divisions, agencies, consultants, contractors and/or subcontractors)?

No  No identifying or potentially identifying information will be released. *Go to B. below.*

Yes  *If* Yes, *indicate the name of the organization(s) and their role(s). If unknown at this time, indicate the type of organization(s):*

|  |
| --- |

*If* Yes, *describe the* ***safeguards*** *(MOU, application, etc.) to ensure that the data will be used* ***solely*** *for the purposes of this request, that no re-release of the data will occur, and that no linkage to other identifiable databases will be attempted or made:*

|  |
| --- |

### Will any data, identifying or non-identifying, from the requested Vital Records be used for legal, administrative, or other actions?

No  *Go to VI. below.*

Yes  *If* Yes, *specify the type(s) of action:*

|  |
| --- |

## **INDIVIDUALS ACCESSING REQUESTED DATA**

Identify every individual who will have access to the requested data including the names of project staff, consultants, contractors, subcontractors, etc. For every individual listed, a data use agreement must be completed. If New York City Vital Records are included in the requested data, a second, separate agreement is required from all users. In identifying individuals authorized to access these data, consider that:

***ONLY THOSE LISTED BELOW AND WHO HAVE SIGNED AND SUBMITTED AN AFFIDAVIT MAY USE OR HAVE ACCESS TO THE REQUESTED DATA, and,***

***ANY UNLAWFUL RELEASE OF CERTIFICATE COPIES OR THE INFORMATION THEY CONTAIN MAY RESULT IN DISCIPLINARY AND/OR LEGAL ACTION.***

| **Name** |  | **Title** |  | **Affiliation** |
| --- | --- | --- | --- | --- |
|  |  |  |  |  |
|  |  |  |  |  |
|  |  |  |  |  |
|  |  |  |  |  |
|  |  |  |  |  |
|  |  |  |  |  |
|  |  |  |  |  |
|  |  |  |  |  |
|  |  |  |  |  |
|  |  |  |  |  |
|  |  |  |  |  |
|  |  |  |  |  |
|  |  |  |  |  |
|  |  |  |  |  |
|  |  |  |  |  |

## **DURATION OF ACCESS TO VITAL RECORDS INFORMATION**

Access to Vital Records is granted for one year from the date of approval.

The maximum length of time that access to a Vital Records data file can be granted under a single application is 3 years***.***

*Indicate the number of years that access to the Vital Records files is requested for the purposes stated in this application.*

| Number of years: |  |
| --- | --- |

Transaction ID Number

Certificate Number

Residence Address-County-MCD

Residence Address-House Number

Residence Address-Direction

Residence Address-Street Name

Residence Address-Street Type

Residence Address-Apartment Number

Residence Address-Zipcode + Ext

Mailing Address-House Number

Mailing Address-Direction

Mailing Address-Street Name

Mailing Address-Street Type

Mailing Address-Apartment Number

Mailing Address-Zipcode + Ext

Mailing Address-MCD Literal

Mother's First Name-Maiden

Mother's Last Name-Maiden

Social Security # Mother

Mother's Med Rec #

Mother's Date of Birth - Day Part

Infant's First Name

Infant's Last Name

Infant's Medical Record #

Infant's Date of Birth - Day Part

Infant's Time of Birth - hour/min

Infant's Time of Birth - am/pm

Father's First Name

Father's Last Name

Social Security # Father

Father's Date of Birth - Day Part

Date of Last Menses - Day Part

Attendant License Number

Municipality of Infant's Birth

Census Tract

Census MCD code

Father's Suffix

NYC Residence

NYC Community District Codes

Social Sec. Release Indicator

Local Register Number

Date Record Filed - month

Date Record Filed - century/year

Date of Receipt in E-Mail - Day Part

Date Entered on Master File - Day Part

Data effective date - Day Part

Social Security number - infant

Infant's Middle Name

Mother's Middle Name-Maiden

Father's Middle Name

OOS State file number

Death Certificate number

Decedent's last name

Injury date - Day Part

Date filed - month/day

Date of death - Day Part

Local register number

Admission date - Day Part

Residence - Street number

Date of birth - Day Part

NYC residence

Decedent's first name

Decedent's middle name

Father's last name

Mother's maiden surname

Injury Date - year

Date of delivery, if recently pregnant

Month of receipt

Medical record number

Certifier of death - license number

Residence - Street name

NYC community district codes

Census MCD

FIPS-zipcode indicator

Residence - Street direction

Residence - street type

Residence - apartment number

MBD-birth certificate number

MBD-year of birth

MBD-state of birth

EDC Case number

Out of state file number
