# Supplementary material for: Severe maternal morbidity: A population-based study of an expanded measure and associated factors
Source: PLoS One. 2017 Aug 7;12(8):e0182343. doi: 10.1371/journal.pone.0182343 (PMC5546569; doi:10.1371/journal.pone.0182343)
Supplement: S1 Table — (DOCX) [file pone.0182343.s004.docx]

| **Characteristic** | **Vaginal deliveries**  N=893,497  Percent of all vaginal deliveries | **Vaginal deliveries with severe maternal morbidity**  N=12,575  Percent of vaginal deliveries with characteristic | **Adjusted odds ratios**  **(95% confidence limits)** |
| --- | --- | --- | --- |
| **Mother’s age**  Less than 20 years old  20 ≤ age ≤ 35  Older than 35 | 64,161( 7.18%)  715,238(80.05%)  114,098(12.77%) | 1,393 ( 2.17%)  9,469 ( 1.32%)  1,713 ( 1.50%) | 0.97 (0.90-1.03)  Referent  1.26 (1.20-1.34) |
| **Race/ethnicity**  White-non-Hispanic  Black-non-Hispanic  Other including multiracial  Hispanic  Missing | 429,579(48.08%)  135,181(15.13%)  111,655(12.50%)  217,039(24.29%)  43( 0.00%) | 4,531 ( 1.05%)  2,631 ( 1.95%)  1,397 ( 1.25%)  4,016 ( 1.85%)  0 ( 0.00%) | Referent  1.08 (1.02-1.15)  1.06 (0.99-1.13)  1.26 (1.19-1.33) |
| **Education**  Less than high school  High school graduate  College graduate or higher  Missing | 190,800(21.35%)  357,108(39.97%)  340,590(38.12%)  4,999( 0.56%) | 3,658( 1.92%)  5,056( 1.42%)  3,738( 1.10%)  123( 2.46%) | 1.16 (1.10-1.21)  Referent  0.95 (0.91-1.00) |
| **Employment during pregnancy**  Yes  No  Missing | 454,567(50.88%)  437,924(49.01%)  1,006( 0.11%) | 5,580( 1.23%)  6,966( 1.59%)  29( 2.88%) | Referent  1.00 (0.96-1.04) |
| **Parity (number of previous births)**  None  One  Two or more  Missing | 384,500(43.03%)  268,454(30.05%)  234,252(26.22%)  6,291( 0.70%) | 6,581( 1.71%)  2,921( 1.09%)  3,005( 1.28%)  68( 1.08%) | 1.56 (1.49-1.63)  Referent  0.98 (0.93-1.03) |
| **Inferred marital status**  Married or living with a partner  Single-divorced or separated  Missing | 639,265(71.55%)  253,743(28.40%)  489( 0.05%) | 7,884( 1.23%)  4,683( 1.85%)  8( 1.64%) | Referent  1.08 (1.03-1.13) |
| **Insurance**  Medicaid  Private insurance  Self-pay or uninsured  Other  Missing | 437,049(48.91%)  406,081(45.45%)  13,422( 1.50%)  34,153( 3.82%)  2,792( 0.31%) | 7,133( 1.63%)  4,567( 1.12%)  316( 2.35%)  504( 1.48%)  55( 1.97%) | 1.19 (1.13-1.25)  Referent  1.41 (1.24-1.59)  1.12 (1.01-1.23) |
| **Nativity: American born**  Yes  No  Missing | 565,620(63.30%)  325,202(36.40%)  2,675( 0.30%) | 7,381( 1.30%)  5,150( 1.58%)  44( 1.64%) | Referent  1.24 (1.18-1.30) |
| **Location: New York City vs Rest of State**  Yes  No | 461,847(51.69%)  431,650(48.31%) | 6,022( 1.30%)  6,553( 1.52%) | 0.51 (0.48-0.53)  Referent |
| **Prepregnancy Body Mass Index (kg/m^2^)**  Underweight (BMI<18.5)  Normal (18.5≤BMI<25)  Overweight (25≤BMI<30)  Obese (30≤BMI<50)  Obese (50≤BMI)  Missing | 45,238( 5.06%)  473,784(53.03%)  209,966(23.50%)  139,961(15.66%)  1,750( 0.20%)  22,798( 2.55%) | 679( 1.50%)  6,320( 1.33%)  2,956( 1.41%)  2,133( 1.52%)  52( 2.97%)  435( 1.91%) | 0.97 (0.92-1.01)  Referent  0.97 (0.92-1.01)  0.96 (0.91-1.02)  0.96 (0.91-1.02) |
| **Weight gain during pregnancy**  20 lbs or less  21-30 lbs  31-40 lbs  41-50 lbs  51 lbs or more  Missing | 224,695(25.15%)  268,799(30.08%)  223,267(24.99%)  96,713(10.82%)  61,347( 6.87%)  18,676( 2.09%) | 3,618( 1.61%)  3,523( 1.31%)  2,821( 1.26%)  1,315( 1.36%)  916( 1.49%)  382( 2.05%) | 1.07 (1.02-1.13)  Referent  0.99 (0.94-1.04)  1.00 (0.94-1.07)  1.01 (0.93-1.09) |
| **Prenatal care**  Intensive  Adequate  Intermediate  Inadequate  No prenatal care  Missing information | 74,556( 8.34%)  499,613(55.92%)  211,134(23.63%)  53,065( 5.94%)  6,927( 0.78%)  48,202( 5.39%) | 1,156( 1.55%)  6,233( 1.25 %)  3,081( 1.46%)  920( 1.73 %)  252( 3.64 %)  933( 1.94 %) | 1.11 (1.04-1.19)  Referent  1.00 (0.95-1.05)  1.03 (0.96-1.11)  1.68 (1.26-2.22)  0.96 (0.89-1.04) |
| **Type of pregnancy**  Singleton baby  Two or more babies  Missing | 887,046(99.28%)  6,065( 0.68%)  386( 0.04%) | 12,128( 1.37%)  443( 7.30%)  4( 1.04%) | Referent  2.98 (2.65-3.35) |
| **Fetal presentation**  Cephalic  Breech  Other  Unknown  Missing | 883,038(98.83%)  2,113( 0.24%)  5,486( 0.61%)  2,454( 0.27%)  406( 0.05%) | 12,362( 1.40%)  123( 5.82%)  67( 1.22%)  19( 0.77%)  4( 0.99%) | Referent  2.07 (1.68-2.55)  0.91 (0.71-1.17)  0.64 (0.40-1.01) |
| **Primary provider for prenatal care**  MD  Clinic  Other  No information  No provider  Indeterminate  Missing | 627,028(70.18%)  200,410(22.43%)  46,847( 5.24%)  8,157( 0.91%)  7,851( 0.88%)  2,783( 0.31%)  421( 0.05%) | 7,080( 1.13%)  4,379( 2.19%)  614( 1.31%)  150( 1.84%)  213( 2.71%)  135( 4.85%)  4( 0.95%) | Referent  1.32 (1.26-1.39)  1.04 ( 0.95-1.14)  0.78 (0.65-0.93)  1.01 (0.82-1.26)  1.04 (0.74-1.46) |
| **Pregnancy hospitalizations**  Yes  No | 16,237( 1.82%)  877,260(98.18%) | 804 ( 4.95%)  11,771( 1.34%) | 1.91 (1.75-2.07)  Referent |
| **Preterm labor**  Yes  No | 104,449(11.69%)  789,048(88.31%) | 3,344( 3.20%)  9,231( 1.17%) | 1.94 (1.85-2.03)  Referent |
| **Delivery hospital: designated level of perinatal care**  1 or 2  3  Regional perinatal center  Non-birthing facility | 309,083(34.59%)  331,928(37.15%)  249,967(27.98%)  2,519( 0.28%) | 2,740( 0.89%)  5,418( 1.63%)  4,407( 1.76%)  10( 0.40%) | Referent  1.95 (1.84-2.06)  1.95 (1.85-2.06)  0.84 (0.45-1.57) |
| **Day of Hospital Admission**  Weekday  Weekend | 651,558(72.92%)  241,939(27.08%) | 9,278( 1.42%)  3,297( 1.36%) | Referent  0.96 (0.92-1.00) |
| **Depression during pregnancy**  Not depressed at all  A little depressed  Moderately depressed  Very depressed  Very depressed and had to get help  Missing | 638,446(71.45%)  134,553(15.06%)  28,887( 3.23%)  4,588( 0.51%)  4,055( 0.45 %)  82,968( 9.29%) | 8,601( 1.35%)  2,045( 1.52%)  462( 1.60%)  116( 2.53%)  81( 2.00%)  1,270( 1.53%) | Referent  1.02 (0.97-1.07)  0.99 (0.90-1.09)  1.43 (1.18-1.73)  1.08 (0.86-1.36) |
| **Cardiac disease** | 3,690( 0.41%) | 153 ( 4.15%) | 2.60 (2.18-3.11) |
| **Renal disease** | 347( 0.04%) | 39 (11.24%) | 3.06 (2.01-4.66) |
| **Musculoskeletal disease** | 2,022( 0.23%) | 58( 2.87%) | 1.66 (0.54-5.09) |
| **Digestive disorder** | 752( 0.08%) | 16( 2.13%) | 1.21 (0.69-2.14) |
| **Diseases of the blood and all blood-forming organs** | 70,961( 7.94%) | 4,103( 5.78%) | 4.95 (4.75-5.15) |
| **Mental disorders** | 35,556( 3.98%) | 861( 2.42%) | 1.18 (1.09-1.28) |
| **Disorders of the central nervous system** | 7,413( 0.83%) | 202( 2.72%) | 1.41 (1.21-1.65) |
| **Rheumatic heart disease** | 261( 0.03%) | 24( 9.20%) | 3.20 (1.88-5.45) |
| **Placentation disorder** | 5,453( 0.61%) | 442( 8.11%) | 3.73 (3.33-4.17) |
| **Chronic hypertension** | 8,509( 0.95%) | 237( 2.79%) | 1.45 (1.25-1.67) |
| **Pregnancy hypertension** | 25,986( 2.91%) | 1,616( 6.22%) | 3.39 (3.19-3.60) |
| **Collagen/vascular disorder** | 257( 0.03%) | 6( 2.33%) | 0.80 (0.24-2.67) |
| **Rheumatoid arthritis** | 782( 0.09%) | 15( 1.92%) | 0.67 (0.20-2.17) |
| **Pulmonary conditions** | 35,435( 3.97%) | 698( 1.97%) | 1.00 (0.92-1.08) |
| **Diabetes** | 3,353( 0.38%) | 118( 3.52%) | 1.06 (0.79-1.41) |
| **Diabetes complicating pregnancy** | 7,190( 0.80%) | 189( 2.63%) | 1.41 (1.13-1.76) |
| **Lupus** | 1,012( 0.11%) | 38(3.75%) | 0.77 (0.25-2.39) |
