# Supplementary material for: Severe maternal morbidity: A population-based study of an expanded measure and associated factors
Source: PLoS One. 2017 Aug 7;12(8):e0182343. doi: 10.1371/journal.pone.0182343 (PMC5546569; doi:10.1371/journal.pone.0182343)
Supplement: S2 Table — (DOCX) [file pone.0182343.s005.docx]

| **Characteristic** | **Cesarean deliveries**  N=459,103  Percent of all cesarean deliveries | **Cesarean deliveries with severe maternal morbidity**  N=21,903  Percent of cesarean deliveries with characteristic | **Adjusted odds ratios**  **(95% confidence limits)** |
| --- | --- | --- | --- |
| **Mother’s age**  Less than 20 years old  20 ≤ age ≤ 35  Older than 35 | 18,123( 3.95%)  344,796(75.10%)  96,184(20.95%) | 1,216( 6.71%)  15,567( 4.51%)  5,120( 5.32%) | 1.16 (1.08-1.25)  Referent  1.11 (1.07-1.15) |
| **Race/ethnicity**  White-non-Hispanic  Black-non-Hispanic  Other including multiracial  Hispanic  Missing | 214,691(46.76%)  80,185(17.47%)  55,624(12.12%)  108,580(23.65%)  23( 0.01%) | 7,216( 3.36%)  6,592( 8.22%)  2,243( 4.03%)  5,851( 5.39%)  1( 4.35%) | Referent  1.60 (1.53-1.68)  1.14 (1.08-1.20)  1.27 (1.21-1.33) |
| **Education**  Less than high school  High school graduate  College graduate or higher  Missing | 76,925(16.76%)  175,878(38.31%)  203,962(44.43%)  2,338( 0.51%) | 5,035( 6.55%)  9,043( 5.14%)  7,561 ( 3.71%)  264(11.29%) | 1.08 (1.04-1.13)  Referent  0.93 (0.89-0.96) |
| **Employment during pregnancy**  Yes  No  Missing | 257,705(56.13%)  200,939(43.77%)  459( 0.10%) | 10,515( 4.08%)  11,323( 5.64%)  65(14.16%) | Referent  1.06 (1.02-1.09) |
| **Parity (number of previous births)**  None  One  Two or more  Missing | 199,425(43.44%)  152,786(33.28%)  104,286(22.72%)  2,606( 0.57%) | 8,868( 4.45%)  5,854( 3.83%)  7,036( 6.75%)  145( 5.56%) | 1.03 (0.99-1.08)  Referent  1.43 (1.37-1.48) |
| **Inferred marital status**  Married or living with a partner  Single-divorced or separated  Missing | 337,765(73.57%)  120,989(26.35%)  349( 0.08%) | 13,717( 4.06%)  8.158( 6.74%)  28( 8.02%) | Referent  1.09 (1.05-1.13) |
| **Insurance**  Medicaid  Private insurance  Self-pay or uninsured  Other  Missing | 189,422(41.26%)  244,524(53.26%)  5,881( 1.28%)  18,137( 3.95%)  1,139( 0.25%) | 11,105( 5.86%)  9,302( 3.80%)  494( 8.40%)  907( 5.00%)  95( 8.34%) | 1.12 (1.07-1.17)  Referent  1.32 (1.18-1.47)  1.12 (1.03-1.21) |
| **Nativity: American born**  Yes  No  Missing | 293,794(63.99%)  163,930(35.71%)  1,379( 0.30%) | 13,491( 4.59%)  8,315( 5.07%)  97( 7.03%) | Referent  1.04 (1.00-1.08) |
| **Location: New York City vs Rest of State**  Yes  No | 219,331(47.77%)  239,772(52.23%) | 11,657( 5.31%)  10,246( 4.27%) | 0.84 (0.81-0.87)  Referent |
| **Prepregnancy Body Mass Index (kg/m^2^)**  Underweight (BMI<18.5)  Normal (18.5≤BMI<25)  Overweight (25≤BMI<30)  Obese (30≤BMI<50)  Obese (50≤BMI)  Missing | 13,525( 2.95%)  195,270(42.53%)  117,923(25.69%)  117,632(25.62%)  2,974( 0.65%)  11,779( 2.57%) | 715( 5.29%)  8,944( 4.58%)  5,498( 4.66%)  5,647( 4.80%)  204( 6.86%)  895( 7.60%) | 1.07 (0.98-1.16)  Referent  0.92 (0.89-0.95)  0.86 (0.83-0.89)  0.86 (0.83-0.89) |
| **Weight gain during pregnancy**  20 lbs or less  21-30 lbs  31-40 lbs  41-50 lbs  51 lbs or more  Missing | 111,282(24.24%)  124,990(27.22%)  112,228(24.45%)  57,244(12.47%)  43,728( 9.52%)  9,631( 2.10%) | 6,630( 5.96%)  5,783( 4.63%)  4,411( 3.93%)  2,299( 4.02%)  2,090( 4.78%)  690( 7.16%) | 1.11 (1.07-1.15)  Referent  0.93 (0.89-0.97)  0.93 (0.88-0.98)  0.99 (0.94-1.05) |
| **Prenatal care**  Intensive  Adequate  Intermediate  Inadequate  No prenatal care  Missing information | 45,794( 9.97 %)  263,250(57.34%)  98,159(21.38%)  24,213( 5.27%)  2,635( 0.57%)  25,052( 5.46%) | 2,029( 4.43%)  10,825( 4.11%)  5,235( 5.33%)  1,590( 6.57%)  351( 13.32%)  1,873( 7.48%) | 1.05 (1.00-1.11) Referent  1.08 (1.04-1.12)  1.16 (1.09-1.24)  1.55 (1.24-1.93)  1.09 (1.03-1.16) |
| **Type of pregnancy**  Singleton baby  Two or more babies  Missing | 437,835(95.37%)  20,984( 4.57%)  284( 0.06%) | 19,762( 4.51%)  2,123(10.12%)  18( 6.34%) | Referent  1.55 (1.46-1.64) |
| **Fetal presentation**  Cephalic  Breech  Other  Unknown  Missing | 400,939(87.33%)  44,486( 9.69%)  11,945( 2.60%)  1,442( 0.31%)  291( 0.06%) | 18,490( 4.61%)  2,558( 5.75%)  768( 6.43%)  68( 4.72%)  19( 6.53%) | Referent  0.99 (0.94-1.03)  1.18 (1.09-1.29)  0.92 (0.71-1.19) |
| **Primary provider for prenatal care**  MD  Clinic  Other  No information  No provider  Indeterminate  Missing | 342,814(74.67%)  98,966(21.56%)  9,570( 2.08%)  2,985( 0.65%)  3,564( 0.78%)  989( 0.22%)  215( 0.05%) | 13,557( 3.95%)  6,883( 6.95%)  684( 7.15%)  233( 7.81%)  394(11.05%)  136(13.75%)  16( 7.44%) | Referent  1.16 (1.12-1.21)  1.32 (1.20-1.44)  0.89 (0.76-1.03)  1.16 (0.98-1.36)  0.99 (0.73-1.35) |
| **Pregnancy hospitalizations**  Yes  No | 14,516( 3.16%)  444,587(96.84%) | 2,011(13.85%)  19,892( 4.47%) | 1.46 (1.38-1.55)  Referent |
| **Preterm labor**  Yes  No | 78,331(17.06%)  380,772(82.94%) | 8,602(10.98%)  13,301( 3.49%) | 1.78 (1.72-1.85)  Referent |
| **Delivery hospital: designated level of perinatal care**  1 or 2  3  Regional perinatal center  Non-birthing facility | 157,689(34.35%)  168,496(36.70%)  131,997(28.75%)  921( 0.20%) | 4,113( 2.61%)  8,489( 5.04%)  9,278( 7.03%)  23( 2.50%) | Referent  1.50 (1.43-1.56)  2.01 (1.93-2.10)  1.12 (0.74-1.71) |
| **Day of Hospital Admission**  Weekday  Weekend | 380,617(82.90%)  78,486(17.10%) | 17,311( 4.55%)  4,592( 5.85%) | Referent  1.18 (1.14-1.22) |
| **Depression during pregnancy**  Not depressed at all  A little depressed  Moderately depressed  Very depressed  Very depressed and had to get help  Missing | 316,831(69.01% )  74,935(16.32%)  16,816( 3.66%)  2,831( 0.62%)  2 421( 0.53%)  45,269( 9.86%) | 14,236( 4.49%)  3,865( 5.16%)  853( 5.07%)  212( 7.49%)  169( 6.98%)  2,568( 5.67%) | Referent  1.04 (0.99-1.10)  0.99 (0.92-1.06)  1.20 (1.02-1.42)  1.20 (1.00-1.45) |
| **Cardiac disease** | 3,112( 0.68%) | 415(13.34%) | 2.29 (2.02-2.59) |
| **Renal disease** | 438 ( 0.10%) | 161(36.76%) | 3.78 (2.98-4.81) |
| **Musculoskeletal disease** | 1,838( 0.40%) | 189(10.28%) | 1.05 (0.53-2.07) |
| **Digestive disorder** | 624( 0.14%) | 34( 5.45%) | 1.17 (0.80-1.70) |
| **Diseases of the blood and all blood-forming organs** | 54,653(11.90%) | 7,550(13.81%) | 3.52 (3.40-3.63) |
| **Mental disorders** | 21,894( 4.77%) | 1,729( 7.90%) | 1.17 (1.10-1.24) |
| **Disorders of the central nervous system** | 5,372( 1.17%) | 473( 8.80%) | 1.43 (1.28-1.59) |
| **Rheumatic heart disease** | 247( 0.05%) | 71(28.74%) | 3.18 (2.30-4.41) |
| **Placentation disorder** | 15,733( 3.43%) | 3,262(20.73%) | 4.02 (3.83-4.22) |
| **Chronic hypertension** | 9,704( 2.11%) | 798( 8.22%) | 1.30 (1.19-1.41) |
| **Pregnancy hypertension** | 32,653( 7.11%) | 4,587(14.05%) | 2.52 (2.42-2.63) |
| **Collagen/vascular disorder** | 319( 0.07%) | 28( 8.78%) | 1.42 (0.71-2.84) |
| **Rheumatoid arthritis** | 672( 0.15%) | 43( 6.40%) | 0.98 (0.50-1.94) |
| **Pulmonary conditions** | 23,629( 5.15%) | 1,722( 7.29%) | 1.13 (1.07-1.20) |
| **Diabetes** | 6,063( 1.32%) | 568( 9.37%) | 0.96 (0.79-1.18) |
| **Diabetes complicating pregnancy** | 8,199( 1.79%) | 695( 8.48%) | 1.24 (1.03-1.48) |
| **Lupus** | 914( 0.20%) | 130(14.22%) | 1.31 (0.68-2.51) |
