# Supplementary material for: Severe maternal morbidity: A population-based study of an expanded measure and associated factors
Source: PLoS One. 2017 Aug 7;12(8):e0182343. doi: 10.1371/journal.pone.0182343 (PMC5546569; doi:10.1371/journal.pone.0182343)
Supplement: S3 Table — (DOCX) [file pone.0182343.s006.docx]

| **Condition** | **2008** | **2009** | **2010** | **2011** | **2012** | **2013** | **P value** | **Change** |
| --- | --- | --- | --- | --- | --- | --- | --- | --- |
| **Acute renal failure** | 0.24 | 0.24 | 0.28 | 0.26 | 0.30 | 0.29 | <.05 | Increase |
| **Cardiac arrest or ventricular fibrillation** | 0.01 | 0.01 | 0.01 | 0.01 | 0.004 | 0.01 | .46 | No change |
| **Heart failure during procedure or surgery** | 0.27 | 0.29 | 0.33 | 0.28 | 0.30 | 0.30 | .20 | No change |
| **Shock** | 0.02 | 0.03 | 0.03 | 0.03 | 0.05 | 0.06 | <.05 | Increase |
| **Sepsis** | 0.05 | 0.07 | 0.06 | 0.05 | 0.06 | 0.07 | .49 | No change |
| **Disseminated intravascular coagulation** | 0.25 | 0.32 | 0.42 | 0.41 | 0.40 | 0.41 | <.05 | Increase |
| **Amniotic fluid embolism** | 0.002 | 0.004 | 0.006 | 0.003 | 0.005 | 0.004 | .33 | No change |
| **Thrombotic embolism** | 0.04 | 0.04 | 0.06 | 0.06 | 0.08 | 0.07 | <.05 | Increase |
| **Puerperal cerebrovascular disorders** | 0.06 | 0.04 | 0.04 | 0.04 | 0.04 | 0.04 | .08 | No change |
| **Severe anesthesia complications** | 0.03 | 0.02 | 0.03 | 0.03 | 0.02 | 0.01 | <.05 | Decrease |
| **Pulmonary edema** | 0.03 | 0.04 | 0.04 | 0.05 | 0.04 | 0.04 | <.05 | Increase |
| **Adult respiratory distress syndrome** | 0.05 | 0.07 | 0.07 | 0.05 | 0.07 | 0.07 | .10 | No change |
| **Acute myocardial infarction** | 0.005 | 0.003 | 0.002 | 0.002 | 0.006 | 0.002 | .53 | No change |
| **Eclampsia** | 0.08 | 0.07 | 0.07 | 0.06 | 0.06 | 0.05 | <.05 | Decrease |
| **Blood transfusion** | 4.22 | 4.44 | 4.85 | 4.94 | 5.24 | 5.52 | <.05 | Increase |
| **Ventilation** | 0.08 | 0.09 | 0.10 | 0.09 | 0.09 | 0.10 | .06 | No change |
| **Hysterectomy** | 0.10 | 0.11 | 0.11 | 0.11 | 0.12 | 0.13 | <.05 | Increase |
| **Anemia including sickle cell** | 7.71 | 8.76 | 10.11 | 10.76 | 11.29 | 12.08 | <.05 | Increase |
| **Intracranial injuries** | 0.002 | 0 | 0.0009 | 0 | 0 | 0.0009 | .23 | No change |
| **Internal injuries of thorax-abdomen-and pelvis** | 0.007 | 0.005 | 0.006 | 0.003 | 0.004 | 0.008 | .79 | No change |
| **Aneurysm** | 0.003 | 0.001 | 0.003 | 0.002 | 0.002 | 0.003 | .62 | No change |
| **Operations on heart and pericardium** | 0.07 | 0.06 | 0.08 | 0.07 | 0.07 | 0.08 | .11 | No change |
| **Cardio monitoring** | 0.03 | 0.03 | 0.06 | 0.02 | 0.01 | 0.01 | <.05 | Decrease |
| **Temporary tracheostomy** | 0.003 | 0.003 | 0.0004 | 0.001 | 0.001 | 0.002 | .41 | No change |
| **Conversion of cardiac rhythm** | 0.01 | 0.01 | 0.01 | 0.01 | 0.01 | 0.01 | .39 | No change |
| **Acute liver failure** | 0.12 | 0.13 | 0.13 | 0.19 | 0.42 | 0.56 | <.05 | Increase |
| **Coma** | 0.004 | 0.001 | 0.0009 | 0.002 | 0.0009 | 0.0009 | .69 | No change |
| **Delirium** | 0.01 | 0.01 | 0.02 | 0.02 | 0.02 | 0.02 | <.05 | Increase |
| **Epilepsy** | 0.003 | 0.003 | 0.002 | 0.004 | 0.003 | 0.002 | .99 | No change |
| **Uterine rupture** | 0.82 | 0.64 | 0.66 | 0.57 | 0.54 | 0.47 | <.05 | Decrease |
| **Urea metabolism disorders** | 0.01 | 0.01 | 0.02 | 0.02 | 0.01 | 0.02 | .41 | No change |
| **Thyrotoxicosis** | 0.20 | 0.21 | 0.24 | 0.23 | 0.24 | 0.27 | <.05 | Increase |
| **Diabetic ketoacidosis** | 0.003 | 0.004 | 0.005 | 0.004 | 0.009 | 0.006 | <.05 | Increase |
| **Thrombocytopenia** | 0.85 | 1.17 | 1.59 | 1.70 | 1.89 | 1.99 | <.05 | Increase |
