# Supplementary material for: Severe maternal morbidity: A population-based study of an expanded measure and associated factors
Source: PLoS One. 2017 Aug 7;12(8):e0182343. doi: 10.1371/journal.pone.0182343 (PMC5546569; doi:10.1371/journal.pone.0182343)
Supplement: S4 Table — (DOCX) [file pone.0182343.s007.docx]

| Covariate | CDC Algorithm  Estimated odds ratio [95% confidence limits] | NY Algorithm  Estimated odds ratio [95% confidence limits] |
| --- | --- | --- |
| 2009 vs 2008 | 0.99 (0.95-1.03) | 0.99 (0.95-1.03) |
| 2010 vs 2008 | 1.26 (1.21-1.31) | 1.25 (1.20-1.30) |
| 2011 vs 2008 | 1.16 (1.12-1.21) | 1.17 (1.12-1.21) |
| 2012 vs 2008 | 1.14 (1.09-1.18) | 1.14 (1.10-1.19) |
| 2013 vs 2008 | 1.14 (1.09-1.18) | 1.14 (1.10-1.19) |
| GIndex: Intensive vs Adequate | 1.08 (1.04-1.13) | 1.07 (1.03-1.12) |
| GIndex: Intermediate vs Adequate | 1.03 (1.00-1.06) | 1.05 (1.02-1.08) |
| GIndex: Inadequate vs Adequate | 1.10 (1.05-1.15) | 1.11 (1.06-1.16) |
| GIndex: No prenatal care vs Adequate | 1.55( 1.30-1.84) | 1.59 (1.34-1.89) |
| GIndex: Missing vs Adequate | 1.04 (0.99-1.09) | 1.04 (0.99-1.09) |
| Weight gain: 20 lbs or less vs 21-30 lbs | 1.10 (1.06-1.13) | 1.10 (1.06-1.13) |
| Weight gain: 31-40 lbs vs 21-30 lbs | 0.97 (0.93-1.00) | 0.96 (0.93-0.99) |
| Weight gain: 41-50 lbs vs 21-30 lbs | 0.97 (0.93-1.01) | 0.96 (0.93-1.01) |
| Weight gain: 51 lbs or more vs 21-30 lbs | 1.02 (0.97-1.07) | 1.01 (0.96-1.05) |
| BMI: Thin vs Normal | 1.07 (1.01-1.13) | 1.07 (1.01-1.14) |
| BMI: Overweight vs Normal | 0.94 (0.91-0.97) | 0.94 (0.91-0.97) |
| 30<=BMI<50 vs Normal | 0.91 (0.88-0.94) | 0.90 (0.87-0.93) |
| 50<=BMI vs Normal | 0.91 (0.88-0.94) | 0.90 (0.87-0.93) |
| Depression: a little vs none | 1.04 (1.00-1.08) | 1.04 (1.00-1.07) |
| Depression: moderately vs none | 0.99 (0.93-1.04) | 0.99 (0.94-1.05) |
| Depression: very vs none | 1.27 (1.10-1.45) | 1.28 (1.12-1.46) |
| Depression: needed help vs none | 1.16 (1.00-1.35) | 1.17 (1.01-1.35) |
| Pregnancy hospitalizations: yes vs no | 1.63 (1.55-1.71) | 1.61 (1.54-1.69) |
| Fetal presentation: breech vs cephalic | 1.00 (0.96-1.05) | 1.01 (0.97-1.06) |
| Fetal presentation: other vs cephalic | 1.22 (1.13-1.32) | 1.20 (1.12-1.30) |
| Fetal presentation: unknown vs cephalic | 0.86 (0.69-1.07) | 0.83 (0.67-1.04) |
| Black not Hispanic vs White not Hispanic | 1.43 (1.38-1.48) | 1.43 (1.38-1.48) |
| Hispanic vs White not Hispanic | 1.29 (1.24-1.33) | 1.29 (1.25-1.34) |
| Other vs White not Hispanic | 1.12 (1.07-1.17) | 1.12 (1.07-1.17) |
| Education: less than HS vs HS graduate | 1.11 (1.08-1.15) | 1.11 (1.08-1.14) |
| Education: College or higher vs HS graduate | 0.93 (0.90-0.96) | 0.93 (0.91-0.96) |
| Age group: less than 20 vs 20-35 | 1.10 (1.04-1.15) | 1.09 (1.04-1.14) |
| Age group: 35 or older vs 20-35 | 1.14 (1.11-1.18) | 1.15 (1.12-1.19) |
| Other vs Private Insurance | 1.14 (1.08-1.22) | 1.13 (1.06-1.20) |
| Medicaid vs Private Insurance | 1.18 (1.14-1.22) | 1.15 (1.11-1.19) |
| Self-pay vs Private Insurance | 1.38 (1.28-1.50) | 1.36 (1.25-1.48) |
| First time mothers vs One previous birth | 1.30 (1.26-1.35) | 1.31 (1.27-1.35) |
| Two or more births vs One | 1.25 (1.22-1.30) | 1.25 (1.21-1.29) |
| Multiple vs singleton pregnancy | 1.74 (1.65-1.84) | 1.73 (1.64-1.82) |
| Employment during pregnancy: no vs yes | 1.04 (1.01-1.07) | 1.03 (1.01-1.06) |
| Nativity: non-US vs US | 1.12 (1.09-1.16) | 1.12 (1.08-1.15) |
| Inferred marital status: no vs yes | 1.11 (1.08-1.15) | 1.09 (1.06-1.12) |
| Delivery method: primary C-section vs vaginal | 2.43 (2.36-2.51) | 2.45 (2.37-2.52) |
| Delivery method: repeat C-section vs vaginal | 2.75 (2.66-2.84) | 2.75 (2.66-2.84) |
| Preterm vs Full term | 1.79 (1.74-1.84) | 1.85 (1.80-1.91) |
| Non-birthing facility vs Level 1 or 2 hospital | 1.08 (0.76-1.52) | 1.01 (0.72-1.43) |
| Level 3 hospital vs Level 1 or 2 | 1.68 (1.62-1.74) | 1.64 (1.59-1.70) |
| RPC vs Level 1 or 2 hospital | 1.94 (1.88-2.01) | 1.98 (1.92-2.05) |
| Primary provider prenatal care: Clinic vs MD | 1.24 (1.20-1.27) | 1.23 (1.19-1.26) |
| Primary provider prenatal care: Other vs MD | 1.13 (1.06-1.20) | 1.10 (1.04-1.17) |
| Primary provider prenatal care: No information vs MD | 0.84 (0.75-0.94) | 0.83 (0.74-0.93) |
| Primary provider prenatal care: No provider vs MD | 1.14 (1.00-1.30) | 1.09 (0.96-1.24) |
| Primary provider prenatal care: NYC indeterminate | 1.07 (0.86-1.35) | 1.05 (0.84-1.31) |
| Day of admission: weekend vs week | 1.08 (1.05-1.11) | 1.07 (1.04-1.10) |
| NYC vs ROS | 0.67 (0.65-0.69) | 0.70 (0.68-0.72) |
| Cardiac disease | 2.20 (1.98-2.44) | 2.38 (2.15-2.64) |
| Renal disease | 3.62 (2.95-4.45) | 3.56 (2.90-4.38) |
| Musculoskeletal disease | 1.21 (0.67-2.20) | 1.18 (0.65-2.14) |
| Digestive disorder | 1.14 (0.83-1.58) | 1.18 (0.86-1.62) |
| Diseases of the blood and all blood-forming organs | 4.04 (3.94-4.15) | 3.98 (3.88-4.08) |
| Mental disorders | 1.16 (1.11-1.22) | 1.18 (1.12-1.23) |
| Disorders of the central nervous system | 1.39 (1.27-1.52) | 1.43 (1.31-1.56) |
| Rheumatic heart disease | 2.85 (2.14-3.80) | 3.17 (2.40-4.18) |
| Placentation disorder | 4.19 (4.01-4.38) | 4.17 (3.99-4.36) |
| Chronic hypertension | 1.30 (1.21-1.40) | 1.35 (1.26-1.45) |
| Pregnancy hypertension | 2.45 (2.36-2.54) | 2.73 (2.63-2.82) |
| Lupus | 1.11 (0.62-1.99) | 1.15 (0.64-2.05) |
| Collagen/vascular disorder | 1.04 (0.57-1.91) | 1.26 (0.69-2.33) |
| Rheumatoid arthritis | 0.88 (0.48-1.62) | 0.88 (0.48-1.61) |
| Diabetes complicating pregnancy | 1.28 (1.11-1.48) | 1.29 (1.12-1.49) |
| Diabetes | 0.92 (0.78-1.09) | 0.95 (0.80-1.12) |
| Pulmonary conditions | 1.09 (1.04-1.15) | 1.09 (1.04-1.15) |
