# Supplementary figures and images for: Severe maternal morbidity: A population-based study of an expanded measure and associated factors
Source: PLoS One. 2017 Aug 7;12(8):e0182343. doi: 10.1371/journal.pone.0182343 (PMC5546569; doi:10.1371/journal.pone.0182343)

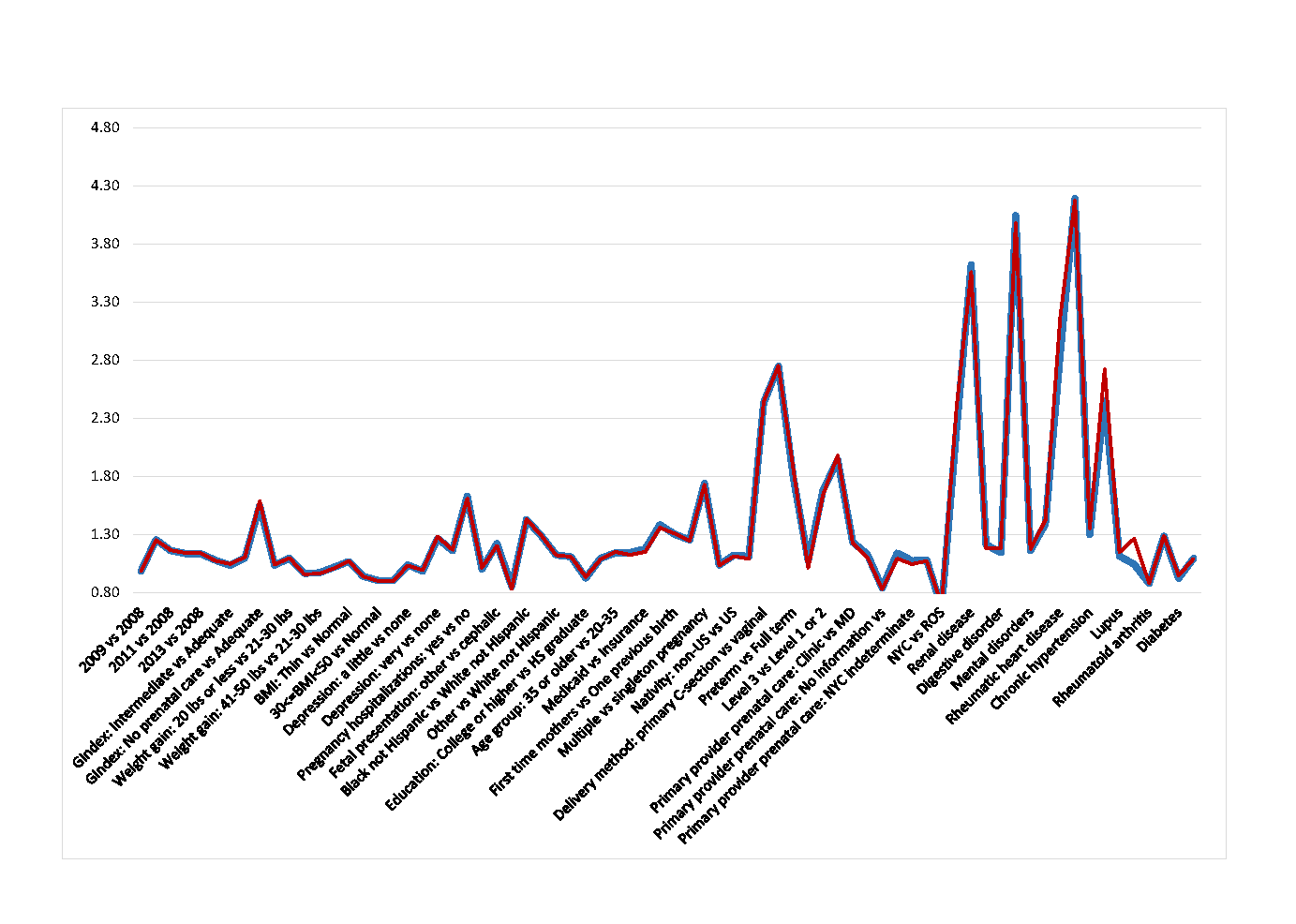

Supplement: S1 Fig — Blue line: CDC SMM measure; red line: NY SMM measure. (TIF) [file pone.0182343.s008.tif]
